# Wrinkling Pickering Emulsions via Endospore Germination

**DOI:** 10.1002/smll.76438

**Published:** 2026-10-29

**Authors:** Yiyao Hu, Flavio Lorez, Jeremy P. H. Wong, Rosa Heydenreich, David S. Kammer, Alexander Mathys, Eleonora Secchi, Lucio Isa

**Affiliations:** ^1^ Laboratory for Soft Materials and Interfaces Department of Materials ETH Zürich Zurich Switzerland; ^2^ Solid Mechanics Department of Civil, Environmental and Geomatic Engineering ETH Zürich Zurich Switzerland; ^3^ Biomatter Microfluidics Department of Civil, Environmental, and Geomatic Engineering ETH Zürich Zurich Switzerland; ^4^ Sustainable Food Processing Laboratory Department of Health Science and Technology ETH Zürich Zurich Switzerland

**Keywords:** bacterial spores, pickering emulsions, responsive materials, wrinkled droplets

## Abstract

Pickering emulsions are stabilized by coating droplet surfaces with solid particles that provide exceptional stability and suppress coalescence. However, these particles are typically inert and lock emulsions into fixed morphologies, limiting their functionality. Creating smart emulsions, which can change their morphology in response to triggers, remains a major challenge. This research introduces an oil‐in‐water Pickering emulsion stabilized by *Bacillus subtilis* endospores that combines long‐term stability with germination‐triggered responsiveness. Upon nutrient exposure, dormant endospores germinate and expand, generating compressive stress on the droplet's surface. In response to this, droplets develop interfacial wrinkles, which correlate with the initial spore surface coverage, with higher coverage producing more pronounced, finer wrinkles. Notably, not only does our emulsion retain stability at room temperature for months, but the endospores remain viable and can be germinated on demand. This work provides a simple route to producing robust, sustainable, and “bio‑actuated” emulsions that undergo controlled interfacial morphological changes.

## Graphical abstract

This study presents a method for creating Pickering emulsions with long shelf life using bacterial endospores as stabilizers. Upon exposure to favorable conditions, spores germinate, swelland trigger wrinkling on droplet surfaces. The resulting patterns depend on initial interfacial coverage, enabling the change of droplet morphology on demand. This platform offers a new way to use bacterial endospores as building blocks to fabricate living materials.

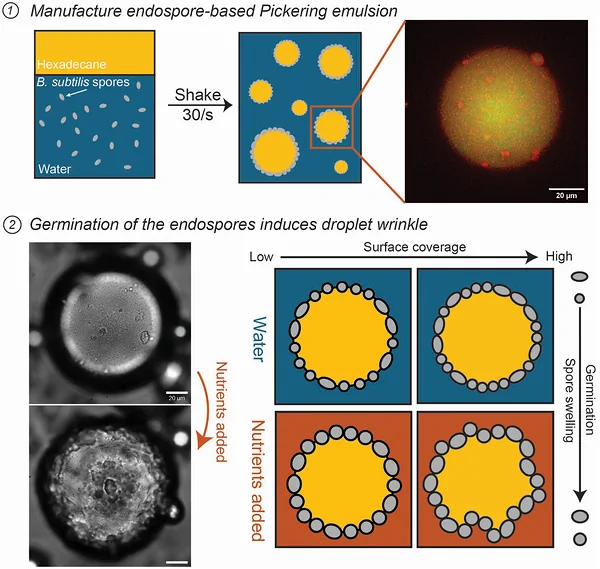

## 1 Introduction

Emulsions are ubiquitous materials that consist of droplets of one immiscible liquid dispersed in another one and are stabilized by emulsifiers. They are widely used across industries, from mining to food and pharmaceuticals, and are also present in everyday products such as mayonnaise and shampoo [1]. While standard emulsions are stabilized by surfactants, droplet coalescence can also be prevented by coating their surfaces with solid micro/nanoparticles, leading to a class of materials known as particle‐stabilized or Pickering emulsions [2]. Even if Pickering emulsions have been known for more than a century, their interest is steadily growing owing to their superior stability, favorable mechanical properties, and the versatility of the choice of materials [3, 4]. These characteristics have enabled advanced applications, for instance, in drug delivery [5, 6], interfacial catalysis [7], oil separation [8], food processing [9, 10], and as templates for porous materials [11]. Commonly, inert particles, such as silica [12], metal oxides [13], clays [14], polymeric particles [15], or micro/nanogels [16, 17], are used as Pickering stabilizers. While these materials are robust, they often lack biocompatibility and biodegradability, and may thus not be suitable for applications involving biomedical and food applications where residual particles can raise safety or regulatory concerns. Only a few biocompatible materials, such as cellulose [18], chitosan [19], and starch [20], have been reported to stabilize Pickering emulsions. In few cases, biologically active particles, such as bacteria [21] and plant spores [22], have also been employed, where the former offer the opportunity to deliver active ingredients produced by the cells.

Differently from plants and fungi, which generate spores as part of their reproduction cycle, bacteria belonging to the phylum Firmicutes form endospores as a survival mechanism under harsh conditions. We will henceforth simply refer to endospores as spores. Several industrially relevant bacteria belonging to the phylum Firmicutes produce endospores, including *Bacillus* species that are widely used for industrial enzyme production (e.g. *Bacillus subtilis*, *Bacillus licheniformis*) and *Clostridium* species that are commonly employed in solvent and biofuel fermentation (e.g. *Clostridium acetobutylicum*). One of the best‐studied non‐pathogenic sporulating organisms is *Bacillus subtilis*. Compared to the vegetative cells, its spores are not only highly resilient to harsh environmental conditions, but also exhibit similar shapes and sizes to colloidal particles, with a major axis length of around 1.2 µm and a minor axis length of around 0.8 µm [23]. The spores remain sensitive to the environment, such as humidity [24] and chemicals [25], while retaining full genetic information of the bacterium. Unlike plant spores that need complex germination conditions, *B. subtilis* spores can, in fact, be germinated and grow into vegetative cells within several hours upon sensing the right germination agent [26]. For example, after detecting nutrient‐rich Tryptic Soy Broth (TSB) medium, germination is triggered. The germinating spores activate enzymes that degrade the cortex and undergo core swelling due to water absorption. The increase in core water content allows active metabolism and macromolecular synthesis, and, with the provided nutrients, germinated spores then outgrow into vegetative cells [26]. Moreover, compared to plant spores, bacterial spores are highly amenable to genetic manipulation, offering an excellent platform for introducing novel functionalities into the resulting biomaterials. In this work, we show that utilizing bacterial spores offers interesting opportunities to obtain large surface area materials with wrinkled surfaces.

Wrinkled surfaces offer high surface‐to‐volume ratios and roughness, and are commonly found across different natural materials and length scales, for example, in skin, intestines, and plant leaves [27]. They also present advantages in industrial applications, such as in biomaterials [28], biosensors [29], catalytic reactors [30], and templates for porous materials [31, 32]. In the case of Pickering emulsions, it has been reported that wrinkling can be induced through several mechanisms. One common approach is to reduce the volume of the dispersed phase, either via evaporation [33, 34] or through the partial solubility of the dispersed phase in the continuous phase [35, 36]. This reduction in the droplet inner volume increases particle packing at the interface and leads to jamming, subsequently causing the interface to buckle and form wrinkles. Wrinkling can also be induced by externally applied interfacial compression, for example, through electric fields [37]. Similarly, wrinkling can be driven by triggering internal particles suspended within the droplet to migrate and adsorb onto an already‐covered interface [34, 38]. In this manuscript, we present an alternative route to achieve droplet wrinkling via a biologically‐driven mechanism without reducing the inner‐phase volume or applying environmental stress.

We use *B. subtilis* spores as emulsifiers to produce oil‐in‐water Pickering emulsions with excellent shelf stability. We then show that, upon nutrient exposure, spores germinate at the liquid interface and increase their size, which induces morphological changes of the droplets through surface wrinkling. As a result, these “bio‐actuated” emulsions can switch from a smooth to a wrinkled interfacial morphology in response to a nutrient trigger. We hypothesize that this transition is driven by compressive stresses generated by spore swelling during germination at the liquid interface and validate our hypothesis by means of direct observations in a Langmuir‐Blodgett trough and via numerical simulations. Both experiments and simulations reveal compression‐induced out‐of‐plane buckling. Finally, we determine that these emulsions not only remain mechanically stable for at least 9 months at room temperature, but that they also fully retain their responsiveness, in the sense that spores can be easily germinated and produce viable cells after prolonged storage at room temperature. Our findings establish bacterial spores as mechanically active, particle‐like building blocks for living materials, and introduce a new class of stable, bio‐actuated Pickering emulsions whose morphology can be changed by biological activity. Additionally, because spores survive harsh processing and long‐term storage while remaining viable, they provide a robust alternative to the direct use of living bacteria in responsive material systems.

## 2 Results

### 2.1 Pickering Emulsions Stabilized by Bacterial Spores

We demonstrate that *B. subtilis* spores can be used to produce stable oil‐in‐water Pickering emulsions. We produced fluorescent *B. subtilis* spores via sporulation and stored them in water in their dormant state until use (see Materials and Methods), allowing us to treat them as inert colloidal particles during emulsion formation and to trigger germination on demand at later times. Their concentration is reported in CFU mL^−1^ (colony‐forming units per mL), which we use here as equivalent to particles mL^−1^. To form the emulsions, we added the oil phase (hexadecane) to a 20 mm NaCl aqueous phase, which contains spores with different spore concentrations ranging from 2 × 10^9^ to 1.2 × 10^10^ CFU mL^−1^, at a 3:7 oil‐to‐water volumetric ratio (Figure 1A). 20 mm NaCl was dissolved in the water to enhance the absorption of spores at the interface by reducing electrostatic barriers. The mixture was then emulsified under constant shaking in a ball milling machine for 15 min. The high energy generated by ball milling leads to the break‐up of the oil phase. Spores then adsorb onto the liquid interface, preventing droplet coalescence, and leading to the formation of stable Pickering emulsions. In traditional Pickering emulsions, a stable droplet size is achieved once all particles are adsorbed to the interface, as the available particles set the total interfacial area that can be stabilized. Consequently, increasing the particle concentration typically decreases droplet size and vice versa, since more particles can stabilize a greater interfacial area [39]. To test whether our system follows this behavior, we prepared emulsions by mixing the oil phase with aqueous spore suspensions at various concentrations and monitored droplet size as a function of spore concentration. No discernible oil phase appeared after 2 days of storage, indicating the formation of a stable emulsion that used up all the available oil (Figure 1B). The droplet diameter correspondingly decreased as a function of increasing spore concentration (Figure 1C). Our emulsification protocol leads to emulsions with polydispersity below 20%, irrespective of the spore concentration. The measured droplet diameters show a similar trend to the theoretical droplet diameter calculated by assuming full surface coverage of spores using the equation mentioned in the Methods section. Notably, the theoretical model predicts that a higher spore concentration would be required to obtain the experimentally observed droplet size, indicating that complete spore coverage of all droplets is not required for emulsion stabilization.

**FIGURE 1:**
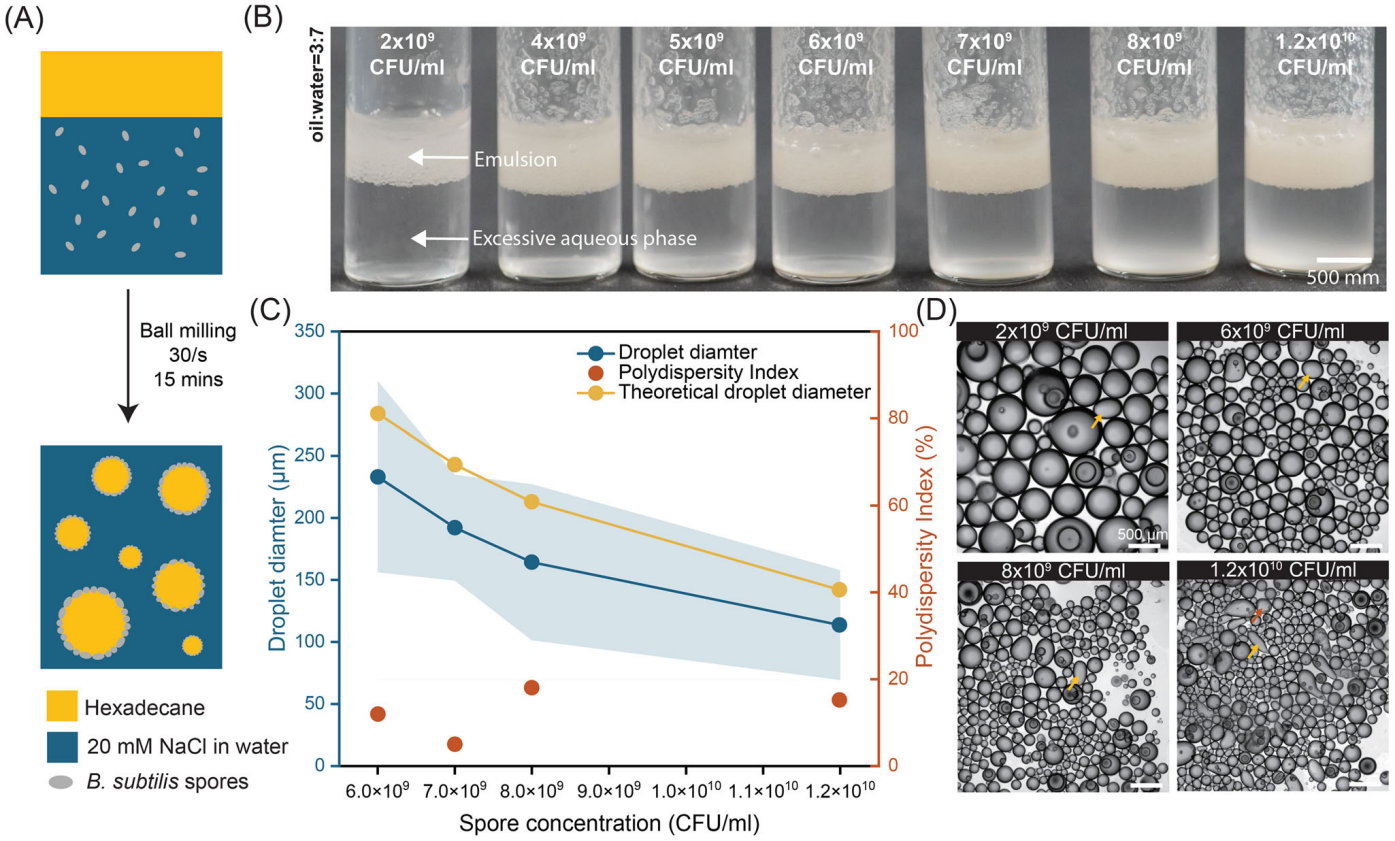
Production and characterization of spore‐stabilized emulsions. (A) Schematic of the fabrication procedure of spore‐based Pickering emulsions. The oil‐to‐water ratio is 3:7. (B) Image of Pickering emulsions containing varying amounts of spores after 2 days, indicating stable emulsions. (C) Impact of initial spore concentration on emulsion droplet size. The shaded region represents the standard error of the mean measured based on at least 70 droplets. Polydispersity was computed based on the diameter of the droplets. (D) Brightfield microscopy images of the emulsion stabilized with varying spore concentrations. The yellow arrow points toward non‐spherical droplets, and the orange arrow points toward a wrinkled droplet from limited coalescence.

By close inspection of the emulsions using microscopy, we observe that non‐spherical droplets coexist with spherical ones across all spore concentrations (Figure 1D). These non‐spherical droplets arise from arrested coalescence [40], indicating that sparsely coated droplets collide and merge during emulsification, forming larger ones with increased surface coverage. Continued coalescence drives the system into a nonequilibrium state, where further relaxation toward spherical shapes is prevented by particle jamming, producing complex droplet morphologies. Similar phenomena have previously been observed in an emulsion stabilized by inert particles [41, 42]. As the spore concentration increases, the fraction of irregularly shaped droplets with high aspect ratios also increases, from almost none in emulsions prepared with 2 × 10^9^ CFU mL^−1^ to approximately 30% in those prepared with 1.2 × 10^10^ CFU mL^−1^. At the highest spore concentration, limited coalescence also leads to the emergence of droplets with a wrinkled surface. We attribute this fact to compressive stresses generated during coalescence, which can drive buckling of the particle‐laden interface. Finally, water‐in‐oil‐in‐water double emulsions were also observed at all spore concentrations, indicating that spores exhibit intermediate wettability, consistent with previous reports for Pickering systems stabilized by particles with moderate hydrophobicity [43, 44].

### 2.2 Spore Size Changes Throughout Germination

To study how germination would affect the Pickering emulsion, we monitored the germination process of fluorescent spores (168mKate2 strain) by incubating them in a liquid culture of TSB over 50 min and observed them on a glass slide after different incubation periods using high‐resolution brightfield and fluorescence microscopy (Figure 2A). Through imaging at various time points, we determined that spore germination is initiated within 10 min upon nutrient supplementation, and it is close to completion at around 50 min, where ∼96% have germinated but have not yet entered the outgrowth phase or formed vegetative cells (Figure S1A). Leveraging brightfield imaging, we successfully sorted and classified spores into two distinct populations throughout the germination process: dormant and germinated spores. Dormant spores appeared dark under brightfield imaging, whereas the germinated spores became transparent with only the outline visible. This difference in appearance is due to the hydration of the germinating spores, which leads to changes in their refractive index [45]. Fluorescence also acts as a proxy for metabolism. The machinery for fluorescence protein synthesis is activated only in metabolically active spores, leading to brighter fluorescence. Conversely, in dormant spores, the fluorescence is caused by the molecules accumulated before germination and remains dim (Figure 2B). The fluorescence images nicely correlate with the brightfield ones, allowing us to distinguish dormant and germinated spores clearly. Moreover, by tracking the germination behavior of genetically engineered *B. subtilis* strain with a fluorescent spore coat in TSB, we observed that during the initial phase of the germination process, the spore coats remain intact, but upon 50 min some coats start to crack open, where the non‐fluorescent vegetative cells then bud out, leaving behind the fluorescent spore coat (Figure 2C). We thus conclude that our spores can readily germinate in liquid TSB culture within roughly 50 min and regrow into cells afterward.

**FIGURE 2:**
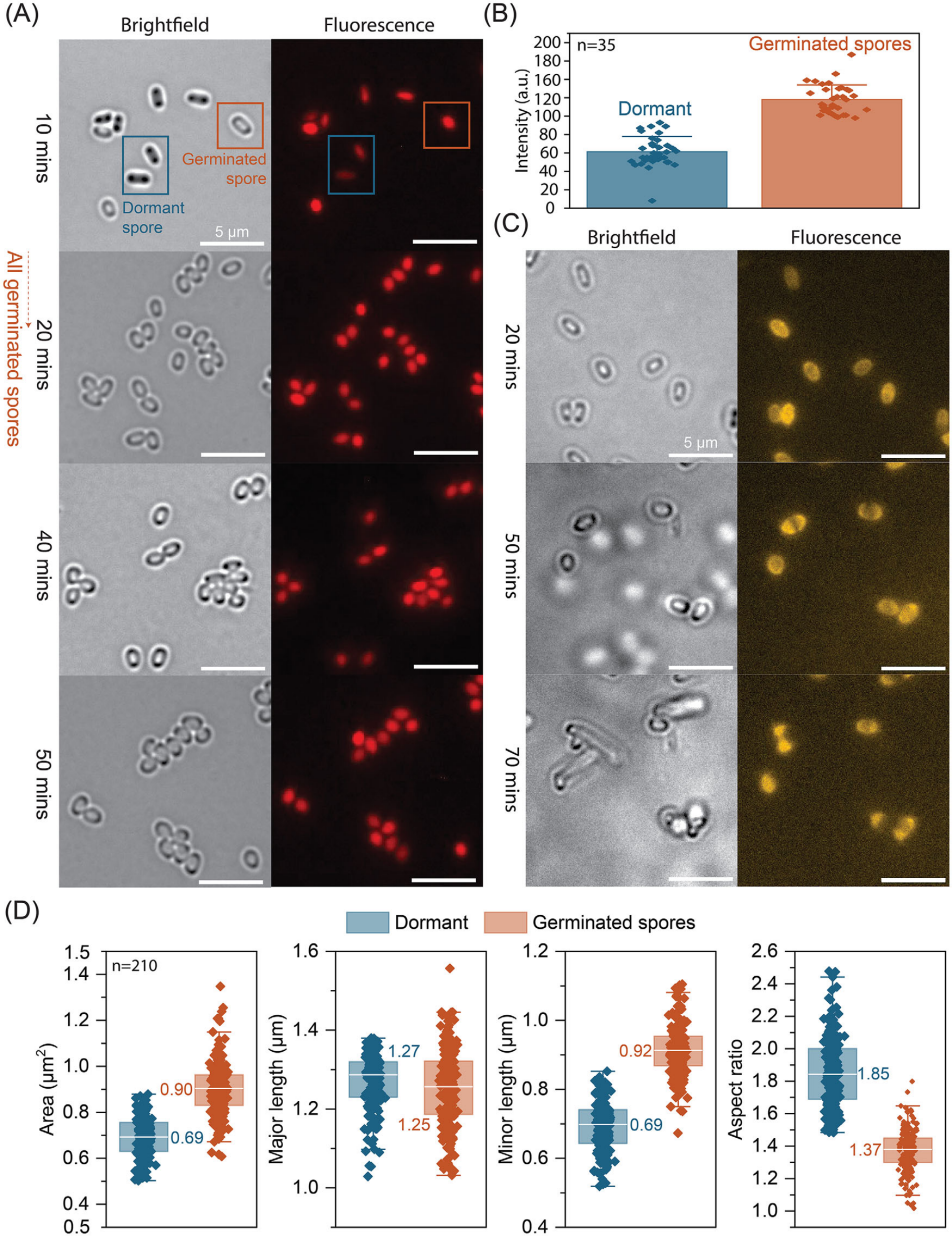
Physiological changes of B. subtilis spores throughout the germination process. (A) Brightfield (left) and fluorescence (right) microscopy images of fluorescent spores 168mKate2 germinated for 10, 20, 40, and 50 min. Germinated vs dormant spores are categorized through brightfield imaging. Fluorescence microscopy images of the same spores, where dormant spores are less bright due to metabolic inactivity. (B) Maximum fluorescence intensity on the germinated and dormant spores, respectively. 35 spores of each category were measured. (C) Brightfield (left) and fluorescence (right) microscopy images of spores with mScarlet‐fused spore coat germinated after 20, 50, and 70 min. We observe that vegetative cells break free from the spore coat at 70 min of incubation from the overlay image. (D) Geometrical differences between dormant and germinated spores. Area, major length, minor length, and aspect ratios were measured for 210 individual spores of each population. The white line within each box represents the median, while the box edges correspond to the 25th (Q1) and 75th (Q3) percentiles. The numbers indicate the mean values.

We noticed that changes in spore size and shape occur already during germination, prior to any cell growth. We thus quantified those by measuring the size of the fluorescently‐labeled 168mKate2 spores, classifying dormant and germinated ones based on the brightfield images. Upon germination, the projected area of the spores increased from 0.69 ± 0.08 to 0.90 ± 0.12 µm^2^, while the major axis length remained nearly constant at around 1.26 µm, the minor axis length increased by 30% from 0.69 ± 0.07 to 0.92 ± 0.07 µm, leading to a decrease in aspect ratio from 1.85 ± 0.21 to 1.37 ± 0.13 (Figure 2D). These changes indicate that spores swell and transition from a more to a less prolate ellipsoidal shape during germination.

### 2.3 Wrinkling of the Emulsion Upon Germination

As in situ changes in the size of emulsion stabilizers have rarely been explored, we sought to utilize the germination‑induced size changes to alter the properties of the emulsion and make the droplets wrinkle. To achieve this, we first generated Pickering emulsions with various spore concentrations as previously described, then proceeded to introduce germination agents to our system and imaged the droplets as a function of time. We assumed that, upon germination, the size change of the spores is similar at the liquid interface and in liquid culture. Similar to spores in TSB culture, we observed spores adsorbed on the surface of the emulsion droplets start germinating within 10 min, leading to dark dots turning gray, and around 86% of the spores at the interface complete their germination within 50 min (see Figure S1). No morphological changes of the droplets were observed with emulsions made with low spore concentration, for example, lower than 2 × 10^9^ CFU mL^−1^ (Figure 3A). By close inspection of emulsions prepared with higher spore concentration, such as 5 × 10^9^ CFU mL^−1^, a subset of droplets showed the emergence of a wrinkled surface after 60 min of germination (Figure 3B). In the emulsion made with an even higher concentration, namely 1.6 × 10^10^ CFU mL^−1^ (Figure 3C‐i), few droplets had a wrinkle pattern already emerging halfway through the germination period (Figure 3C‐ii). These droplets wrinkle continuously as the spores germinate, leading to a more pronounced pattern (Figure 3C‐iii). These observations lead us to hypothesize that the morphological evolution of the droplets during the germination process is strongly correlated with their initial surface spore coverage, where droplets with an initially high spore surface coverage develop wrinkles, and those with relatively low spore coverage remain smooth. We believe that this observation is due to the increase in their cross‐section at the interface as spores germinate, leading to compression of the interfacial layer. Depending on the surface coverage, expansion of the spores may be accommodated either by available space or a build‐up in internal stress. Once full coverage is reached, stress build‐up causes out‐of‐plane buckling and thus causes wrinkling. To confirm the 3D morphology of the wrinkles, we further imaged these germinated wrinkle droplets via confocal microscopy. Z‐stack images show a rough outline and a deformation of the droplet (Figure 3D).

**FIGURE 3:**
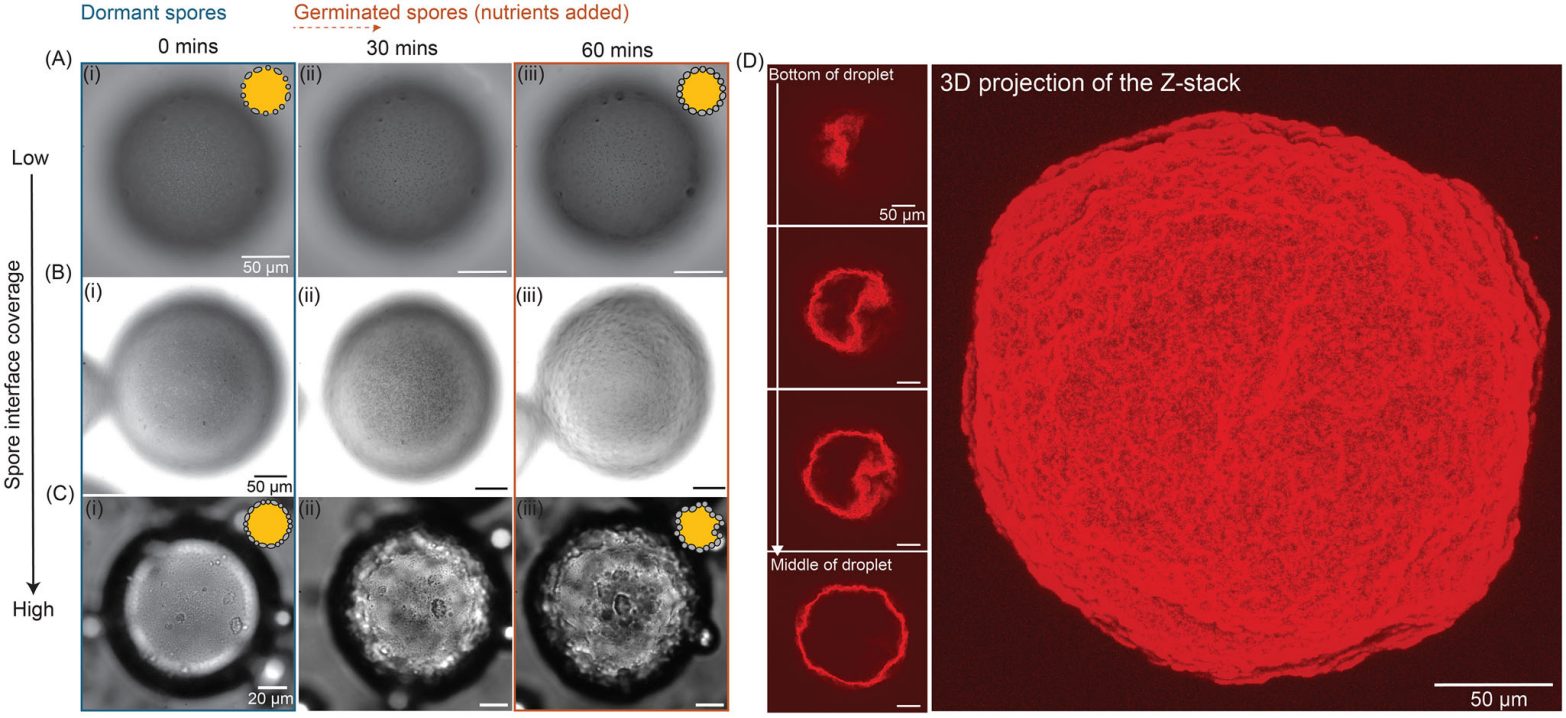
Germination‐induced wrinkling of emulsion droplets. Microscope images of emulsion droplets stabilized by low surface coverage (A), medium surface coverage (B), and high surface coverage (C) spores during germination. We observe wrinkling of medium‐coverage droplets at 60 min, while wrinkling already happens halfway through germination in high‐spore‐coverage droplets. Dark spots left at 60 min represent superdormant spores [46]. (D) Z‐stack and the 3D projection of the z‐stack from a confocal fluorescence image of a germinated wrinkled droplet.

Together, these results indicate that spore swelling during germination increases the packing on the droplet surface. Depending on the initial surface coverage, germination can push the monolayer toward maximal packing, beyond which an increase in the volume of swollen spores drives out‐of‐plane buckling.

### 2.4 Langmuir‐Trough Mimicking the Wrinkling of the Emulsion

Although we observe a clear correlation between initial surface coverage and wrinkling in emulsion droplets, the surface coverage cannot be precisely controlled during emulsification. To further characterize this correlation, we thus performed Langmuir–Blodgett trough experiments to generate spore monolayers of various initial interfacial coverages and systematically test the effect of initial surface coverage on wrinkling behavior, which we quantified by brightfield microscopy.

First, we spread the spores at the hexadecane–MilliQ water interface. Then, using the Langmuir trough, we compressed the monolayer by closing the barriers until buckling occurred. At the onset of buckling, we recorded the corresponding trough area and defined this as the maximum coverage before buckling (ϕ*). We then generated starting interfaces with lower interfacial coverages by opening the barriers to predefined relative positions, allowing controlled comparison across different initial conditions. Figure 4A–C shows microscopy data of monolayers with 80%ϕ*, 90%ϕ*, and 100%ϕ* spore coverage, respectively. We then proceeded to germinate these spore monolayers and monitored the wrinkle formation at the interface via microscopy. Our data suggest that with 80%ϕ* coverage, around 82% of spores germinated after 108 min of incubation, and no wrinkles were observed (Figure 4D) ‐ out of these 108 min, approximately 1 h was required for the temperature to increase from room temperature to 35°C. At 90%ϕ* coverage, after the same incubation time, variations in brightness intensity were observed on the monolayer due to out‐of‐plane buckling (Figure 4E). Upon buckling, the monolayers are displaced orthogonally to the imaging plane, resulting in intensity variations. In Figure 4E,F, dark regions represent wrinkles bending downward into the water phase, while the bright regions represent upward buckling toward the oil phase. These results were further confirmed by z‐stack videos of 100%ϕ* monolayer coverage after 108 min incubation (Movie S1). For 100%ϕ* coverage, a more pronounced wrinkle pattern developed (Figure 4F). Upon monitoring the interface as a function of time, we observe that wrinkling happens on a similar time scale as observed for the emulsions (Figure S2). These data suggest that the initial spore concentration determines the appearance of wrinkling, and, as a result, interface coverage lower than 80%ϕ* does not generate enough stress for wrinkling.

**FIGURE 4:**
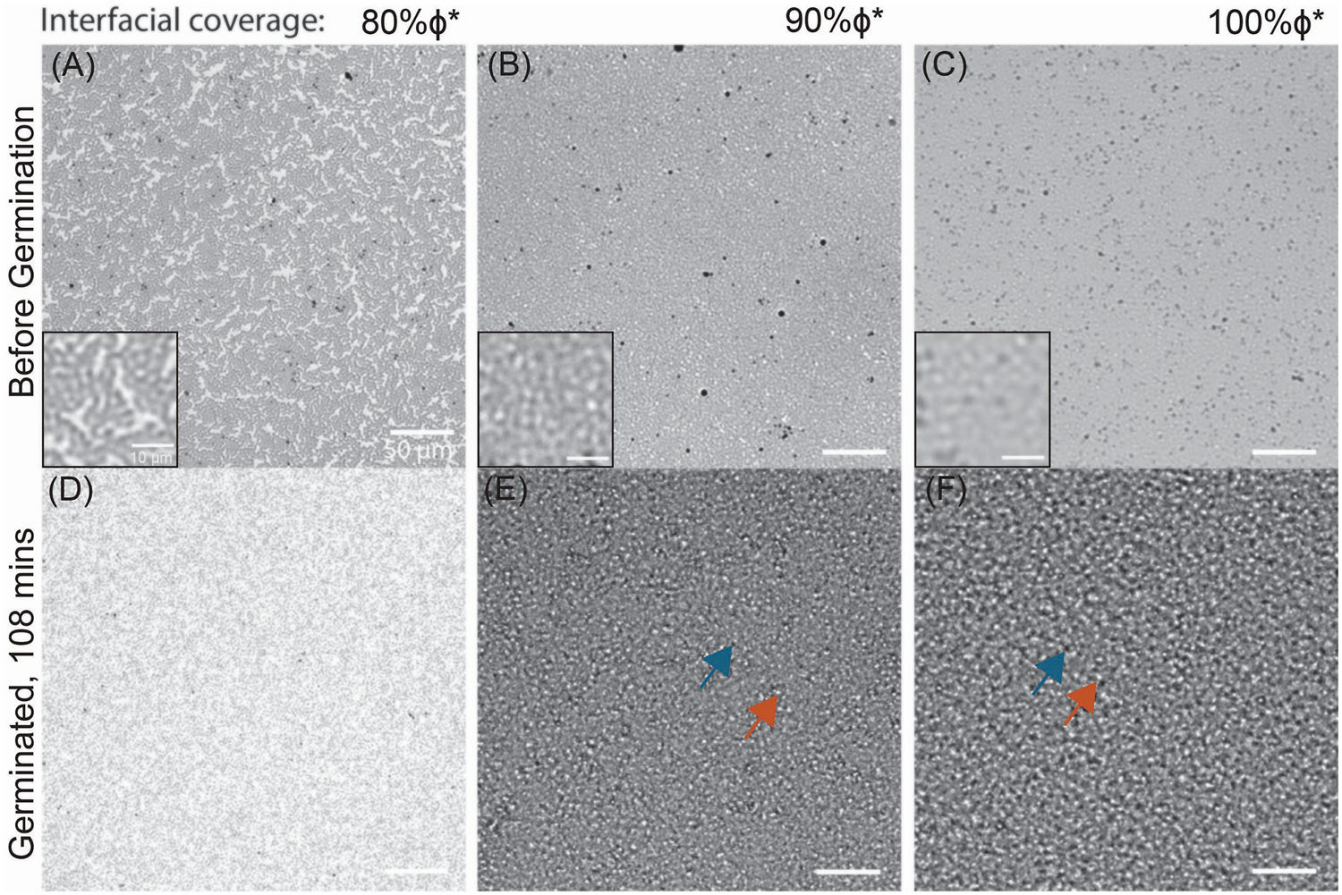
Germination‐induced wrinkling of the spore monolayer in the Langmuir trough. Brightfield microscopy images of spore monolayer with 80%ϕ*, 90%ϕ*, and 100%ϕ* interfacial coverage, respectively, before (A–C) and after 108 min germination (D–F). Zoomed‐in images show we successfully produced monolayers with various initial spore coverages. The ellipsoidal shape of the spores results in capillary attraction between spores, leading to aggregation instead of homogeneous distribution across the interface [47]. After 108 min of germination, we observed wrinkles for 90%ϕ* and 100%ϕ* interfacial coverage conditions. The blue arrow shows a low‐intensity region, where the monolayer bends toward the lower water phase, while the orange arrow indicates the bending toward the upper oil phase.

Further mechanistic insight is gained by examining a localized region on the spore monolayer and applying particle image velocimetry (PIV) analysis to the microscopy images before and after germination (Figure 5Ai,ii). The analysis shows coexistence of expansion and contraction areas around the wrinkles (Figure 5Aiii–v and see Movie S2 for additional data). These data thus suggest that spores swelling during germination causes expansion, which generates in‐plane compression. To relieve this stress, the monolayer undergoes out‐of‐plane buckling, providing a larger surface area for the swollen spores. When in‐plane compression exceeds the combination of the out‐of‐plane bending energy and restoring forces, wrinkling becomes energetically favorable [48]. In this argumentation, we assume that spores are irreversibly trapped within the interface. Considering a contact angle of 90 degrees for simplicity, the trapping energy of a single dormant spore can be calculated as,

$$E=\pi ab\gamma_{ow}$$

where a is the semi‐short axis, b is the semi‐long axis of the spores, and γ_ow_ is the interfacial tension between oil and water phases. At 35°C, the trapping energy is in fact ∼4 × 10^6^ k_B_T and ∼5.2 × 10^6^ k_B_T for the dormant and germinated spore at the hexadecane‐TSB interface, respectively, indicating no spores will be detached from the liquid interface during the experiment. In addition, we quantified the spore number density of the monolayer using microscopy images. Coverage levels before and after germination showed no statistically significant difference, confirming that no desorption of spores occurred from the liquid interface during wrinkling (Figure S3). Moreover, the absence of spores in the lower TSB phase after wrinkling, as observed directly in the z‐stack video (Movie S1), confirms that no spores desorb from the interface due to compression.

**FIGURE 5:**
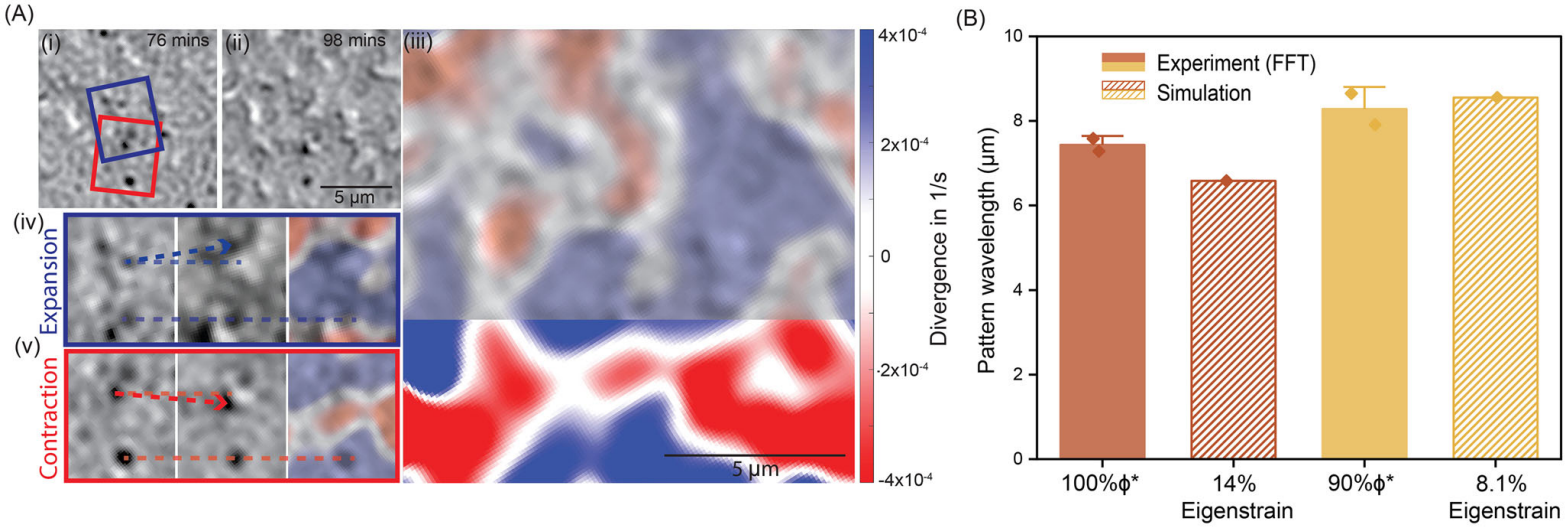
Mechanistic understanding of the wrinkling monolayer. (A) PIV analysis of a wrinkled spore monolayer with 100%ϕ* initial coverage. The zoomed‐in brightfield microscope image shows the monolayer at 76 min (i) and 98 min (ii) after adding TSB and heating, which corresponds to approximately 16 min and 38 min after germination. A clear wrinkled groove can be recognized from these two pictures. We applied PIV analysis to these images and found both expansion and contraction regions (iii). The zoomed‐in regions confirm that positive divergence corresponds to expansion (iv) while negative divergence shows contraction (v) caused by spore swelling. (B) Comparison between simulation and experimental results of the wrinkle wavelength. Experimental wrinkle pattern wavelength with 90%ϕ* and 100%ϕ* initial interfacial coverage was analyzed using radial intensity FFT. Analysis was performed using two independent replicates on each experimental condition. Detailed analysis of the experimental wavelength determination through FFT can be found in Figure S2. The shaded bars represent the simulated pattern wavelength with 14% and 8.1% applied eigenstrain, which corresponds to the eigenstrain calculated based on the swelling of spores and assuming that the monolayer contains the maximum packing fraction of spores. Both simulation and experimental results showed the same trend, where higher surface coverage leads to a smaller wavelength.

To support our understanding of the out‐of‐plane buckling of the spore monolayer, we compared the wrinkling behavior from our experiments with numerical simulations. Based on the projected area change of the germinated spores, we calculated the eigenstrain for 100%ϕ*, 90%ϕ*, and 80%ϕ* spore coverage. Assuming the crystalline maximum packing fraction of 0.91 as representing 100%ϕ* spore coverage and isotropic swelling [49], the resulting eigenstrains for 100%ϕ*, 90%ϕ*, and 80%ϕ* spore coverage are 14%, 8.1%, and 2.3%, respectively (see Method section and Supplementary Text for detail). We then applied these eigenstrains to an elastic particle raft model [50]. The simulation shows that 14% and 8.1% eigenstrain produce wrinkles with eigenvalues of −2.167 and −0.817, respectively, whereas 2.3% eigenstrain generates eigenvalues an order of magnitude lower than the 8.1% case at −0.081. These results are consistent with the previous experimental observations, where pronounced wrinkles were observed in high interfacial coverage conditions, e.g. 90%ϕ* and 100%ϕ* cases. The simulation also shows that the wrinkle wavelength decreases with increasing eigenstrain, i.e. it reveals that the wrinkle wavelength for 14% eigenstrain is smaller than that of 8.1% (Figure 5B). Simulations conducted over a broader range of eigenstrain values further validate this trend (Figure S4). Meanwhile, the normalized out‐of‐plane displacement maps of the simulations resemble the wrinkle patterns observed in experimental microscope images (Figure S5).

To offer a close comparison to the experiments, we identified the wrinkle peaks after 108 min incubation via thresholding the microscopy images at varying intensity levels. We then measured the distance between the wrinkled peaks using images with different thresholds and selected the range of threshold values that resulted in a consistent distance to extract the pattern wavelength via FFT in a robust fashion (Figure S6). The results reported in Figure 5B show close agreement of the trend with the simulations, where the pattern wavelength with 100%ϕ* spore coverage is smaller than that of 90%ϕ*, with 7.4 ± 0.2 and 8.3 ± 0.5 µm, respectively. This agreement implies that our spore monolayer acts similarly to a thin elastic shell. Differences in values between simulation and experiments likely arise because we estimate eigenstrain assuming isotropic in‐plane expansion. However, individual spores swell anisotropically. Moreover, the experimental monolayer never reaches true maximal packing, so changes in projected spore area do not quantitatively translate into the estimated compressive eigenstrains. Furthermore, the simulation assumes the particle raft acts as a continuous thin elastic sheet [50], while real rafts also exhibit granular characteristics [51].

To further confirm that the wrinkling mechanism is driven by spore germination, and to rule out the possibility of wrinkling caused by the exchange of TSB to Milli‐Q water, we replaced the Milli‐Q water with TSB and maintained the Langmuir trough at 4°C. Under these conditions, no wrinkles were observed, suggesting that nutrient addition alone, including any associated osmotic effects or changes in interfacial properties, is insufficient to induce wrinkling (Figure S7). To assess possible changes in surface properties from germination, we performed additional zeta potential measurements. The zeta potential of dormant and germinated spores did not show significant differences, with a slight decrease of about 6% in the absolute value, indicating that surface charge modifications are not the primary driver of the wrinkling process (Figure S8). While hydrophobicity changes between germinated and dormant spores could affect the contact angle of spores at the interface, causing the wrinkling, only minor changes in hydrophobicity have been observed between them in *Bacillus* [52].

Overall, we can conclude that the wrinkling is caused by spore expansion, and by tuning the initial spore coverage, we can affect the compressive stress and thus can change the wavelength of the wrinkling pattern and the surface‐to‐volume ratios.

### 2.5 Long‐Term Stability and Viability of Emulsion

Upon exploring the tunability of our bio‐actuated emulsion system, we turned our attention to its stability, as parameters such as shelf life, storage, and long‐term viability of the spores are crucial for future application considerations. To test the long‐term stability of our emulsions, we stored them for 280 days at room temperature. Our sample showed no signs of phase separation, as seen in Figure 6A. We then measured the droplet size of the 280‐day‐old sample through fluorescence imaging. Our results indicate that our oil‐in‐water droplets retained their size and shape upon more than 9 months of storage (Figure 6B). Furthermore, the calculated emulsification index of the 280‐day‐old emulsion was not significantly different from the fresh emulsion, indicating superior, long‐term stability (Figure 6C). Finally, we tested the germination capability and viability of the spores by exposing the long‐term stored emulsion to germination agents. Emulsion stored for 280 days shows no sign of wrinkling prior to the introduction of germination conditions (Figure 6D). This observation indicates that dormant spores do not spontaneously generate interfacial wrinkles, even after prolonged storage. After introducing nutrients and 35°C temperature, we observed a transition from smooth droplets to wrinkled droplets, demonstrating that spores retained their germination capability (Figure 6E). The emulsions were then incubated overnight, the turbidity developed in the TSB indicating outgrowth and proliferation of vegetative cells. Overall, these results demonstrate that our emulsions are not only stable over long periods of time, but that the biological functionality and responsiveness of their “bio‐actuated” components are also preserved.

**FIGURE 6:**
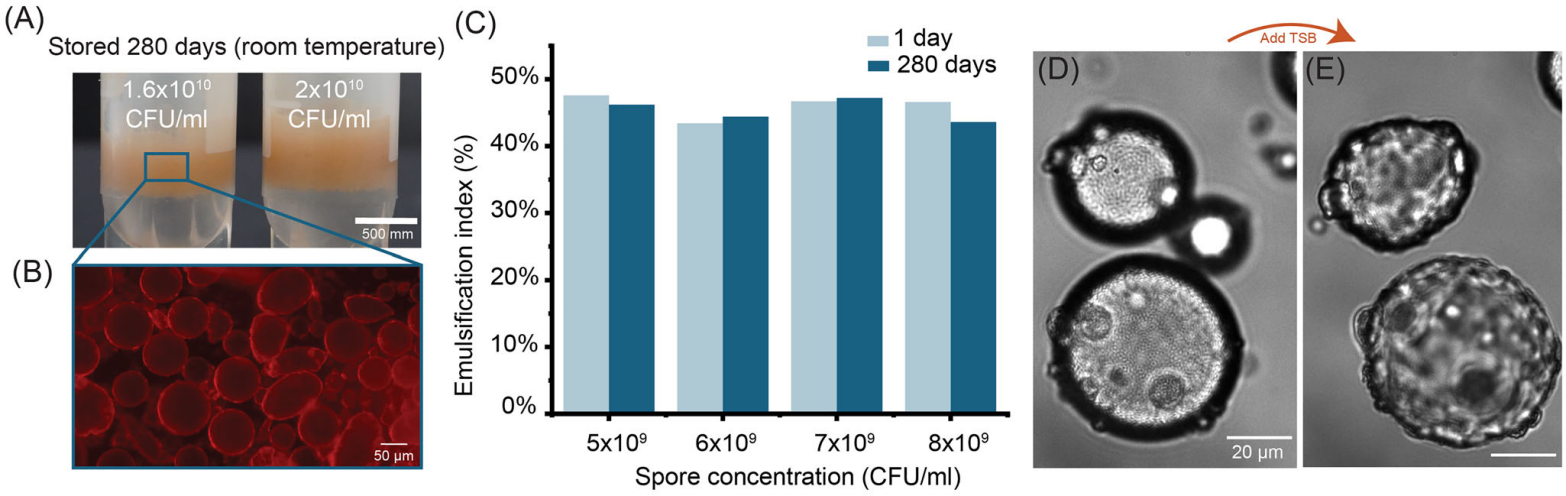
Long‐term stability and viability of emulsion. (A) Digital photographs of emulsions, made with 1.6 × 10^10^ and 2 × 10^10^ CFU mL^−1^, respectively, and stored for 280 days at room temperature. (B) Fluorescence microscope image of the emulsion droplets from an emulsion made with 1.6 × 10^10^ CFU mL^−1^ spore concentration and stored for 280 days. (C) Emulsification index of emulsions between 1 day and 280 days. Microscopy image of the emulsion droplets made with 1.6 × 10^10^ CFU mL^−1^ spores concentration stored for 280 days before (D) and after germination (E).

## 3 Conclusion

In this work, we explore bacterial spores as an alternative to conventional inert particles and living bacteria for stabilizing Pickering emulsions. As spores are resistant, biodegradable, highly responsive, and can grow into bacteria via germination, they provide a robust alternative to approaches that use live bacteria, which require strict manufacturing conditions to prevent bacterial inactivation. More importantly, germination provides a mechanism to actively modify spore size at the interface and thus to mechanically reshape the droplets after emulsion formation. In our system, we use nutrient‐induced germination to trigger spore swelling and induce wrinkling of the droplet interface. However, germination can be activated through a range of stimuli, including specific nutrients such as L‐alanine or L‐valine, as well as physical triggers such as isostatic high pressure [25, 26], offering multiple routes to achieve morphological transformations. This approach, therefore, provides a general strategy to trigger the morphology of Pickering emulsions using biologically active particles. Moreover, we envisage achieving precise wrinkle control via emulsions with a controlled initial coverage, which can, for instance, be obtained by microfluidics [53].

To date, most bacteria‐stabilized emulsions rely on lyophilized microorganisms. Those have been reported to show phase separation within days to 1 month, indicating only limited stability [54, 55]. Conversely, our spore‐stabilized emulsions maintain both viability and stability over many months. Moreover, unlike emulsions stabilized by living bacteria, our emulsions do not require the presence of nutrients to ensure cell viability [56]. Thus, the combination of exceptional stability and the ease of storage makes spores highly suitable as emulsion stabilizers for applications that require long shelf life and minimal handling, such as applications with biological samples that remain active in nutrient‐poor conditions and without the need for refrigeration, as in probiotics [57], biopesticides [58], plant promoters [59], and aquaculture [56]. While the spores used in this work did not display engineered surface functionalities, the characterization of their interfacial behavior provides a foundation for future studies of modified spores. In particular, protein engineering strategies that enable surface display could be explored to introduce additional functionalities of spore‐based materials. Moreover, spores can be regenerated repeatedly via sporulation of outgrown cells, which makes endospore‐based materials, such as our emulsion, potentially recyclable.

Overall, this study presents a new strategy for manufacturing living materials by employing bacterial spores in an analogous way to inert particles for emulsion stabilization. The mechanism by which compressive stresses, leading to wrinkling, are generated, is distinct from previous studies reporting that wrinkling can arise from droplet shrinkage, particle jamming, or externally imposed interfacial compression. In contrast, here the driving force lies in the biological activity of the stabilizing particles themselves, where germination‐induced swelling increases the effective packing fraction and leads to buckling under self‐generated compression. This example of a biologically actuated Pickering emulsion in which stabilizing particles actively reshape the interface through a physiological process opens exciting opportunities for future work to design adaptive, sustainable, and multifunctional living materials through the utilization of bacterial endospores.

## 4 Experimental Section/Methods

### 4.1 Strains and Culture Media

The strains *B. subtilis* 168mKate2 and *B. subtilis* 168 amyE::PcotYZ_cotZ‐mScarlet were used in this work. Strain *B. subtilis* 168mKate2 was obtained from Bacillus Genetic Stock Center (BGSCID 1A1205). Strain *B. subtilis* 168 amyE::PcotYZ_cotZ‐mScarlet was produced in our lab. For details regarding strains and plasmids, refer to Tables 1 and 2. Unless indicated, bacteria were grown in TSB at 37°C.

**TABLE 1:** *Bacillus* strains and plasmids used in this work.

Reagent type	Background	Designation	Source	Description
Strain	*E. coli* DH5α	DH5α WT	Thermo Fisher	DH5α wildtype strain for plasmid storage
Strain	*B. subtilis* 168	LI051‐*B. subtilis* 168 WT	Alexander Mathys, ETH Zurich	Wildtype strain
Strain	*B. subtilis* 168 amyE::P_cotYZ__cotZ‐mScarlet	LI092 – *B. subtilis* 168 amyE:: P_cotYZ__cotZ‐mScarlet	This study	amyE insertion of cotZ‐mScarlet fusion protein in *B. subtilis* 168. Regulated by the cotYZ promoter
Strain	*B. subtilis* 168 amyE::(Pveg(+1/+8)_R0mKate2)::spc trpC2	LI036 – *B. subtilis* 168mKate2	Bacillus Genetic Stock Center	amyE insertion of mKate2 fusion protein in *B. subtilis* 168. Regulated by enhanced Pveg(+1/+8) promoter. Carries spectinomycin resistance and containing the trpC2 mutation.
Plasmid	pUC18t Mini Tn7 Gm Ptet_mScarlet	pUC18t Mini Tn7 Gm Ptet_mScarlet	[60]	Plasmid containing mScarlet sequence used in this study
Plasmid	pBS1C	pBS1C	[61]	amyE insertion empty plasmid backbone, confers chloramphenicol resistance
Plasmid	pJW033	pBS1C_P_cotYZ__cotZ‐mScarlet	This study	Plasmid for amyE insertion of P_cotYZ__cotZ‐mScarlet in *B. subtilis* 168

**TABLE 2:** Primers used in this work.

Primer number	Primer name	Description	Primer sequence (5′ → 3′)
oJW027	pBS1C‐PCot_ FOR	Amplification of CotYZ promoter	CCTTTTTTGCCGGACTGCAGAGCAACAAATACACTCGTAGC
oJW011	pBS1C‐PCot_ REV	Amplification of CotYZ promoter	CTTGATGTTTTCTGGCTCATTGATTTCAGCTCCTTCTTTATAGG
oJW012	cotZ_FOR	Amplification of CotZ gene	ATGAGCCAGAAAACATCAAGC
oJW013	cotZ‐REV	Amplification of CotZ gene	ACCGGATCCGCCACCATGATGATGTGTACGATTGATTAATC
oJW009	cotZ_mScarlet_FOR	Amplification of mScarlet	GGTGGCGGATCCGGTGTGAGCAAGGGCGAGG
oJW028	cotZ_mScarlet_REV	Amplification of mScarlet	CCTAGAAGCTTATCGAATTCTTACTTATACAGTTCATCCATGCC

### 4.2 Construction of Spore Coat Mutants in *B. Subtilis* 168

Plasmids for genomic integration into *B. subtilis* were constructed as previously described [61]. Briefly, the plasmid backbone pBS1C was digested with the restriction enzymes PstI (New England Biolabs) and EcoRI (New England Biolabs). The CotYZ promoter and CotZ genes were then amplified by PCR using *B. subtilis* 168 genomic DNA as the template. The mScarlet gene was amplified from a previously described plasmid [60] via PCR. The resulting PCR products and digested backbone were then analyzed on a 1% agarose gel and gel‐extracted (Qiagen). The final insertion plasmids were then assembled by combining the digested backbone, PCotYZ promoter, CotZ, and mScarlet via Gibson Assembly (New England Biolabs). The assembled plasmid was then transformed into *E. coli* DH5α via heat shock and selected on Lysogeny Broth (LB) plates containing 100 µg ml^−1^ of the antibiotic carbenicillin (Carb). Several colonies were then picked and cultured overnight in LB‐Carb media, where plasmids were then isolated. The isolated plasmids were then sequenced prior to transformation in *B. subtilis*. For transformation of plasmids into *B. subtilis*, an overnight culture of *B. subtilis* 168 was diluted 1000x in Spizizen Minimal Medium (SMM) [62] with supplemented L‐tryptophan. The culture was then grown to an OD of roughly 0.8, where 10 uL of the plasmid of interest was then added and incubated for 50 min at 37°C under constant shaking. 2×TY media was then added to the tube and further incubated for 40 min at 37°C under constant shaking. The resulting culture was then harvested and plated on LB plates containing 5 µg mL^−1^ of chloramphenicol (Cm). Colonies were then selected and characterized via sporulation experiments.

### 4.3 Growth Media for Transformation

All *E. coli* strains were cultured in normal LB media with the corresponding antibiotics required for selection. *B. subtilis* were pre‐incubated in SMM prior to transformation. SMM was prepared as previously described [62]. Briefly, 1 L of Spizizen Salts (SP Salts) solution was prepared by dissolving 14 g K_2_HPO_4_, 6 g of KH_2_PO_4,_ and 1 g of Trisodium citrate. The solution was then sterilized via autoclaving. 10 mL of SMM was then prepared by mixing 9.2 mL of SP Salts with 400 µL of 50% (w/v) glucose, 200 µL of 5% (w/v) cas‐amino acids, 5 µL of 22 mg mL^−1^ ammonium ferric citrate, 50 µL of 40% (w/v) potassium glutamate, 100 µL of 50 mg ml^−1^ L‐tryptophan and 30 µL of 1 m MgSO_4_. 2xTY medium for cell rescuing was prepared by dissolving 16 g of tryptone, 10 g of yeast extract, and 5 g of NaCl in 1 L of MilliQ water, followed by autoclaving.

### 4.4 Sporulation of the *B. Subtilis* Spores

Sporulation of *B. subtilis* was performed as previously described, with minor modifications [63]. Briefly, *B. subtilis* was first grown on a tryptic soy agar (TSA) plate at 37°C overnight. Single colonies were then selected and inoculated into 10 mL of TSB for overnight growth at 37°C under constant shaking. 1 mL of the overnight culture was then transferred into a tube containing 9 mL of fresh TSB, where the culture was then grown to a final OD ∼1.8–2.0. Resulting cultures were then spread onto Difco sporulation medium (DSM) plates containing 8 g L^−1^ DifcoTM nutrient broth, 1 g L^−1^ potassium chloride, 0.12 g L^−1^ magnesium sulfate heptahydrate, 1mL L^−1^ of 1M calcium nitrate, 0.01 m manganese(II) chloride and 1 mM iron(II) sulfate, as well as 15 g L^−1^ agar powder, air‐dried, and incubated at 37°C over the course of 4 days for 168mKate2 and 5 days for the spore coat fusion mutant with mScarlet.

*B. subtilis* spores were then harvested from the DSM plates via transfer of the spore layer into 30 mL of ice‐cold MilliQ water. The harvested spores were then rigorously washed with sterilized Milli‐Q water and centrifuged to ensure the removal of residual nutrients and vegetative cells. Resulting spore solutions may be stored at 4°C for up to 14 days, where then the washing procedure must be repeated to ensure the purity of the solution.

### 4.5 Germination of the Spores and the Size Analysis

Spore germination was performed by introducing spores to a 30 g L^−1^ TSB solution. The suspensions were then incubated at 37°C, 250 rpm. To visually follow the spore physiology throughout the germination procedure, small aliquots of the suspension were taken every 10 min and imaged with a 100× magnification Plan Fluor oil immersion objective in both brightfield and fluorescence mode. Image analysis was then performed to analyze the changes in spore physiology throughout germination. Briefly, fluorescence images were locally thresholded to compute spore sizes, while brightfield images were used to confirm germination by assessing the spore color. Near the end of the germination process, the germination becomes very slow in both TSB liquid culture and on the emulsion droplet surface. Less than ∼0.05% of spores germinated from 50 to 60 min. In this study, we take 50 min as the time when germination is complete.

### 4.6 Fabrication of Spore‐Based Emulsion

Emulsions were prepared by first mixing spores of varying concentrations with 700 µL of 20 mM NaCl solution, where NaCl was used to enhance the absorption of spores at the interface. 300 µL of filter‐sterilized hexadecane was then added to the mix, where the mixture was then emulsified using a ball milling machine (Retsch MIXER MILL MM 400) operating at 30 s^−1^ for a duration of 15 min without milling balls. Previous study has reported the affinity of *B. subtilis* spores to hexadecane, thus hexadecane was chosen as the oil phase [64]. All the liquids were filtered with 0.2 µm pore size filter. The prepared emulsions were then stored at room temperature.

The theoretical spore concentration required to cover oil droplets was estimated from the balance between droplet interfacial area and the projected cross‐sectional area of spores at the oil–water interface, following the standard Pickering emulsion model [65], assuming monolayer adsorption, a contact angle of 90°, oil‐to‐water ratio of 3:7, and the maximum packing of spores at 0.91 [49], *a* is the semi‐major axis of spores, *b* is the semi‐minor axis, and *R* is the droplet radius.

$$\mathrm{Spore}\;\mathrm{conc}\mathrm{entr}\mathrm{ation}\;=\frac{8.19}{7\pi \mathrm{abR}}\;$$

### 4.7 Microscopy Images of the Emulsion Droplets

Microscopy images of emulsions were taken with custom observation cells. We fabricated the cells as follows: we drilled a hole of 1 cm diameter in a 1 mm thick glass slide using a CNC machine. A cover glass was then fixed using UV glue to the bottom of the hole. The TSB solution or MilliQ water was first added to the well, followed by the emulsion droplets. A second coverslip was then placed on top, which was held in position by capillary forces. The setup was imaged using a Nikon Eclipse Ti2‐E inverted microscope.

To monitor the germination of the droplets, the setup was sealed with Parafilm in order to minimize water evaporation while allowing gas exchange during incubation, and then was imaged over time with a temperature control maintained at 35°C. To further check the spore surface coverage of the droplets, the same custom cell was also used for imaging under a confocal microscope (Nikon Ti2 Eclipse) with a Spinning Disk Confocal system.

### 4.8 Langmuir‐Blodgett Trough Experiment

The Langmuir trough (KSV NIMA Trough KN 3003) was first thoroughly cleaned with ethanol and MilliQ water three times prior to experiments. The aqueous subphase of the trough was then filled with filtered MilliQ water, and the oil phase with filtered hexadecane. *B. subtilis* 168mKate2 spores with a concentration of 3.75 × 10^9^ CFU mL^−1^ were first washed three times in MilliQ, and then resuspended in ethanol as the spreading agent. The interface was then cleaned using an aspirator prior to spreading the spore. The spore suspension used for spreading was first vortexed for 5 min and sonicated for 1 min to eliminate possible aggregates. 4 mL of the spreading solution was then injected onto the interface at a speed of 1.6 mL h^−1^ using a syringe pump (NE‐1000 New Era Systems, Inc.) through a PTFE tube (0.03 mm inner diameter) positioned directly at the interface. The resulting interface was then imaged using an upright Nikon LV‐FMA microscope with a 20× magnification objective.

After spreading, the barrier was closed at 10 mm min^−1^ until monolayer bending was observed. Then the barriers were reopened at the same speed until bending disappeared. The barrier position was recorded with the trough area noted as A_0_ and the spore coverage noted as ϕ*. The position was verified by repeated opening and closing cycles three times. The barrier position for different surface coverage was calculated based on the trough area. The barriers were then opened at the same speed to certain positions until reaching surface coverage of 80%ϕ* and 90%ϕ*.

After the desired barrier position was reached, 10 mL MilliQ water was removed from outside the trough each time and replaced by the same amount of concentrated TSB. These processes were repeated until reaching the total TSB concentration of 30 g L^−1^. The thermal circulator connected to the trough was then turned on and set at 35°C. The temperature stabilized after around 1 h, as indicated by the consistent focal plane of the interface. After 1 h of stabilization, z‐stack brightfield images (2 µm steps, total depth 80 µm) were acquired every 2 min for 2 h using an upright microscope.

### 4.9 FFT Analysis

The Fast Fourier Transform was first performed on individual images to monitor the emergence of the wrinkle pattern during germination and quantify the wrinkle pattern size, where the mean value of the intensity at different distances from the center was calculated as the radial intensity. The resulting signal was then smoothed using a Savitzky–Golay filter and normalized to the intensity at a wavenumber of 0.001.

### 4.10 PIV Analysis

The particle image velocimetry analysis was performed in PIVlab MATLAB [66]. Using 3‐pass analyses with interrogation areas of 64, 32, and 20 px, respectively. The step size for each pass was set to half of the corresponding interrogation area. Vector fields were subsequently validated using velocity‐based validation with a standard deviation filter threshold of 8 and a local median filter threshold of 3. Interpolation of missing vectors was enabled. Finally, the divergence map was computed based on the vectors and time intervals.

### 4.11 Simulation

A linear stability analysis of a thin elastic shell about its equilibrium state was performed. The estimation of the eigenstrain ɛ of the 100%ϕ* spore coverage conditions assumes maximum packing fraction of the spores and ignores the aspect ratio changes during germination for simplicity. The estimated eigenstrain is calculated as below, where 0.69 and 0.9 are the spore projected area before and after germination. *B* denotes the coverage fraction relative to ϕ*.

$$\;\frac{0.9-\frac{0.69}{B}}{\frac{0.69}{B}}=2\varepsilon +\varepsilon^{2}$$

A detailed simulation method can be found in the Supporting Text.

## Author Contributions

conceptualization: Y.H., E.S., L.I. investigation: Y.H., E.S., L.I. methodology: Y.H., F.L., J.P.H.W. software: Y.H., F.L. visualization: Y.H., F.L. resources: Y.H., J.P.H.W., R.H. supervision: E.S., L.I., D.S.K., A.M. data curation: Y.H., F.L. formal analysis: Y.H., F.L. writing – original draft: Y.H., F.L., J.P.H.W. writing – review and editing: Y.H., L.I., E.S., J.P.H.W. funding acquisition: L.I., D.S.K. validation: Y.H., F.L., J.P.H.W. project administration: Y.H.

## Funding

ETH Research Grant (24‐1 ETH‐020).

## Conflicts of Interest

The authors declare no conflicts of interest.

## Supporting information




**Supporting File 1**: smll76438‐sup‐0001‐SuppMat.pdf.

**Supporting File 2**: smll76438‐sup‐0002‐MovieS1.avi.

**Supporting File 3**: smll76438‐sup‐0003‐MovieS2.avi.

## Data Availability

All data needed to evaluate the conclusions in the paper are present in the paper and/or the Supplementary Materials.
