# Supplementary material for: Wrinkling Pickering Emulsions via Endospore Germination
Source: Small. 2026 Oct 29;22(63):e76438. doi: 10.1002/smll.76438 (PMC13755993; doi:10.1002/smll.76438)
Supplement: Supplementary file 1 [file SMLL-22-e76438-s003.pdf]

Supporting Information

Wrinkling Pickering Emulsions via Endospore Germination

Yiyao. Hu, Flavio. Lorez, Jeremy P H. Wong, Rosa. Heydenreich, David. Kammer, Alexander. Mathys, Eleonora. Secchi[4], Lucio. Isa[*]*

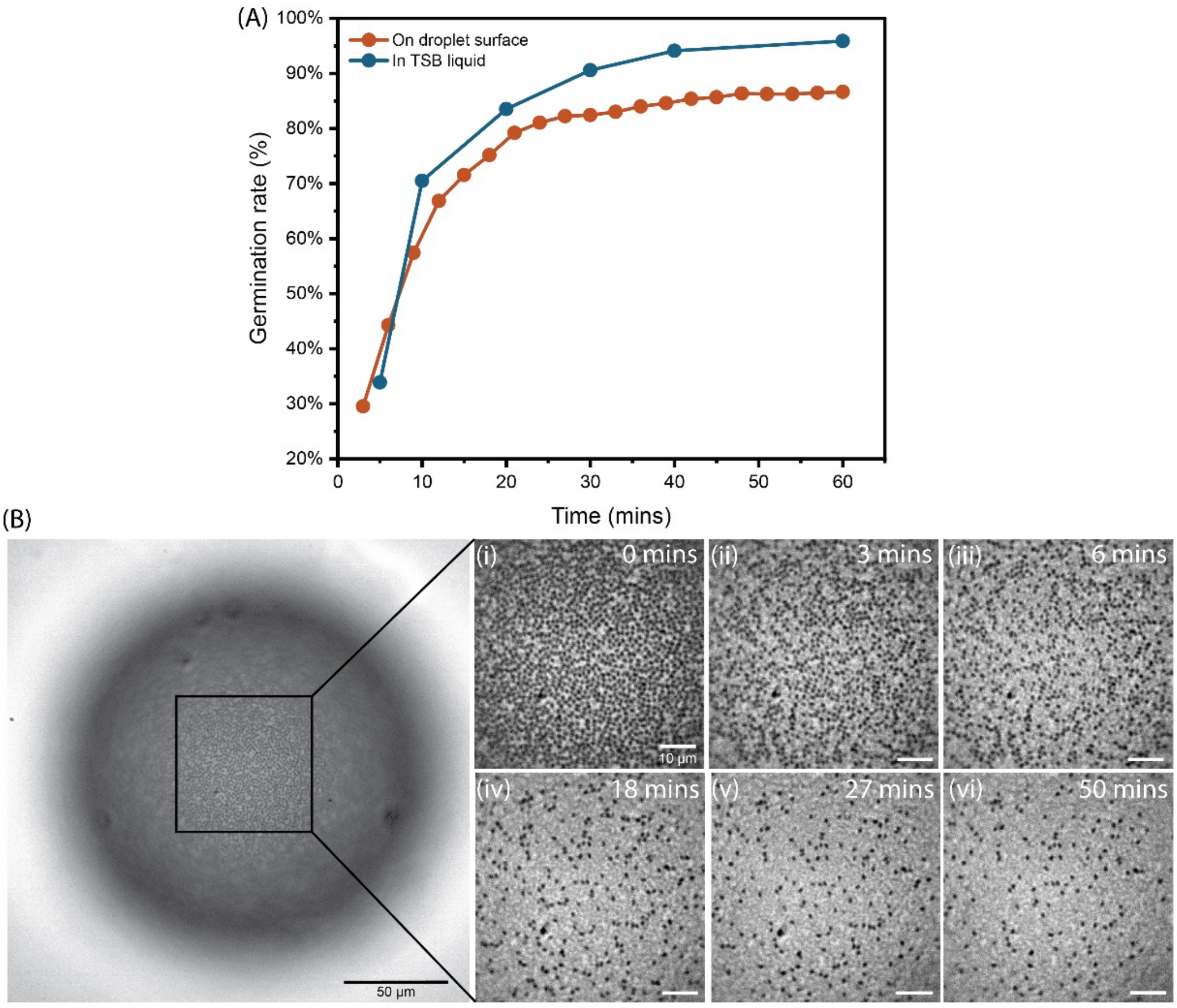

Figure. S1. The germination of spores. (A) The germination rate of 168mKate2 spores in 250 rpm shaking liquid TSB culture (at least 170 spores are analyzed) and on the surface of the droplet (according to B) at 35 °C. The slightly lower germination rate on the droplet surface could be due to slightly different physicochemical microenvironments between TSB and at the liquid interface. **(B)** The germination of 168mKate2 spores on emulsion droplets. Zoomed microscope images (i-vi) of spores on the surface of the emulsion droplet monitored for over 50 mins. The black dots indicate dormant spores, while the grey dots indicate germinated spores after hydration.

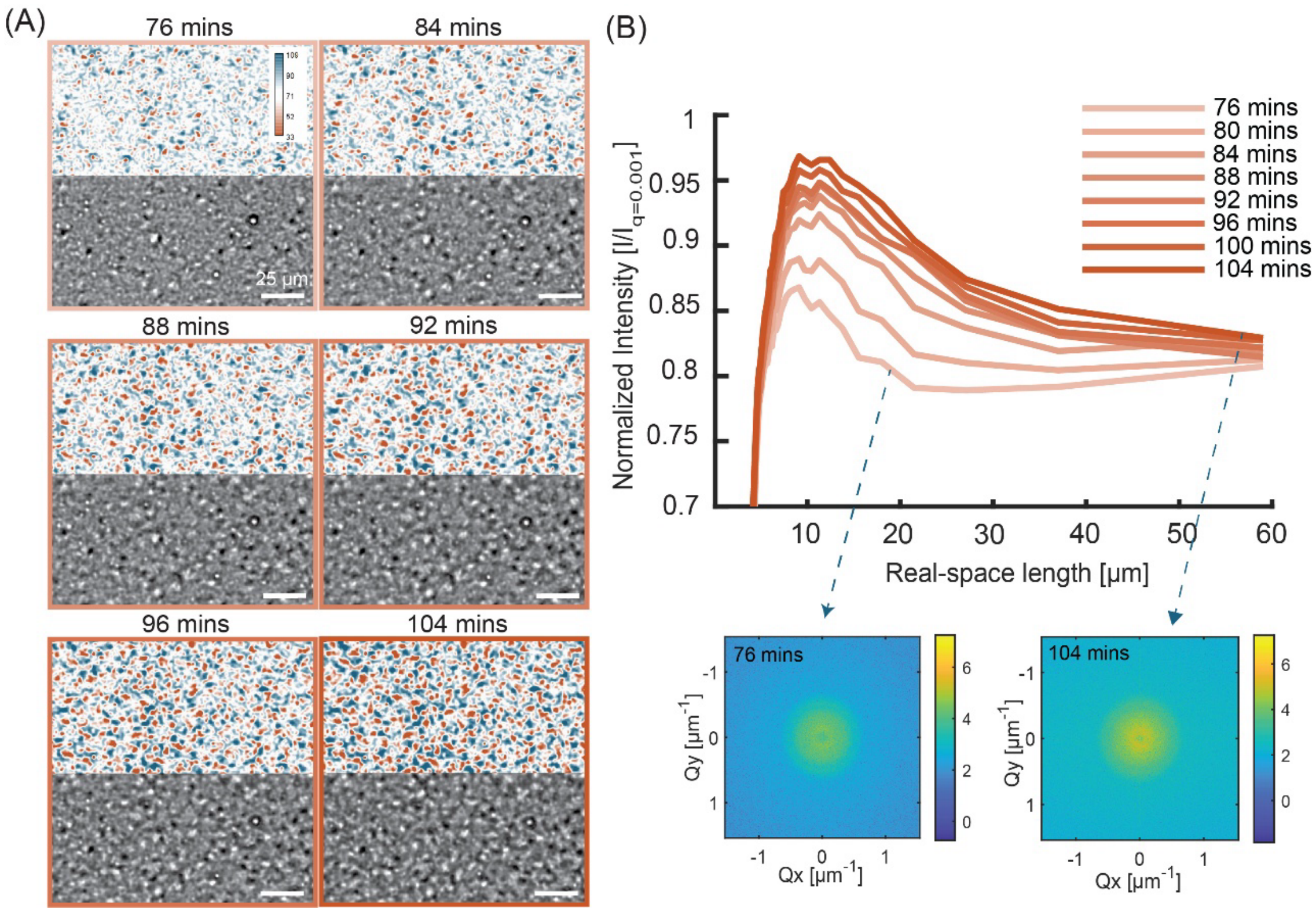

Figure. S2. Monitoring the germination of spore monolayers over time. (A) The brightfield microscopy images monitor the evolution of spore monolayers with 100%ϕ* interfacial coverage during germination. The top part is the heat map converted from the brightfield image for better vision, where orange represents high intensity, and blue represents low intensity. The bottom part of the image is the original brightfield images where we observed the emergence of the bright and dark spots over time. The wrinkling pattern appears at around 76 mins, roughly corresponding to 16 mins after the temperature in the trough reaches homogenously 35 °C. **(B)** Radial intensity of FFT performed on a time-lapse of images with 100%ϕ* interfacial coverage during germination. A consistent FFT peak can be found during germination, and FFT images at 76 mins and 104 mins show a distinct ring, indicating the formation of wrinkles. The intensity of the peak increases, suggesting wrinkles got more pronounced over time. The signal was smoothed using Savitzky-Golay Filters, then normalized with the intensity value when the wavenumber equals 0.001.

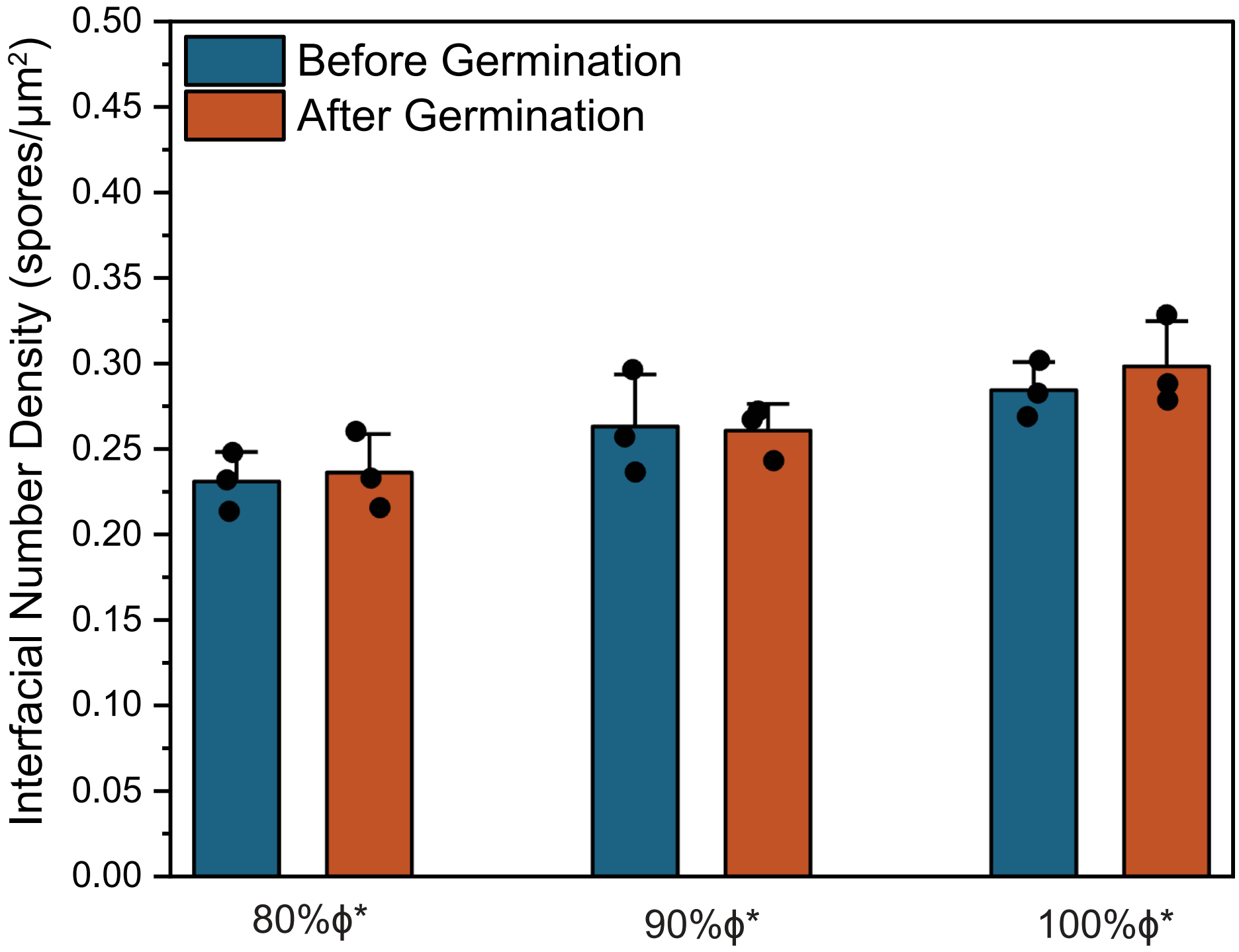

Figure. S3. 2D interfacial spore number density of the monolayer before and after germination with different initial spore coverage. Three areas of 50x50 pixels were chosen from each case for analysis.

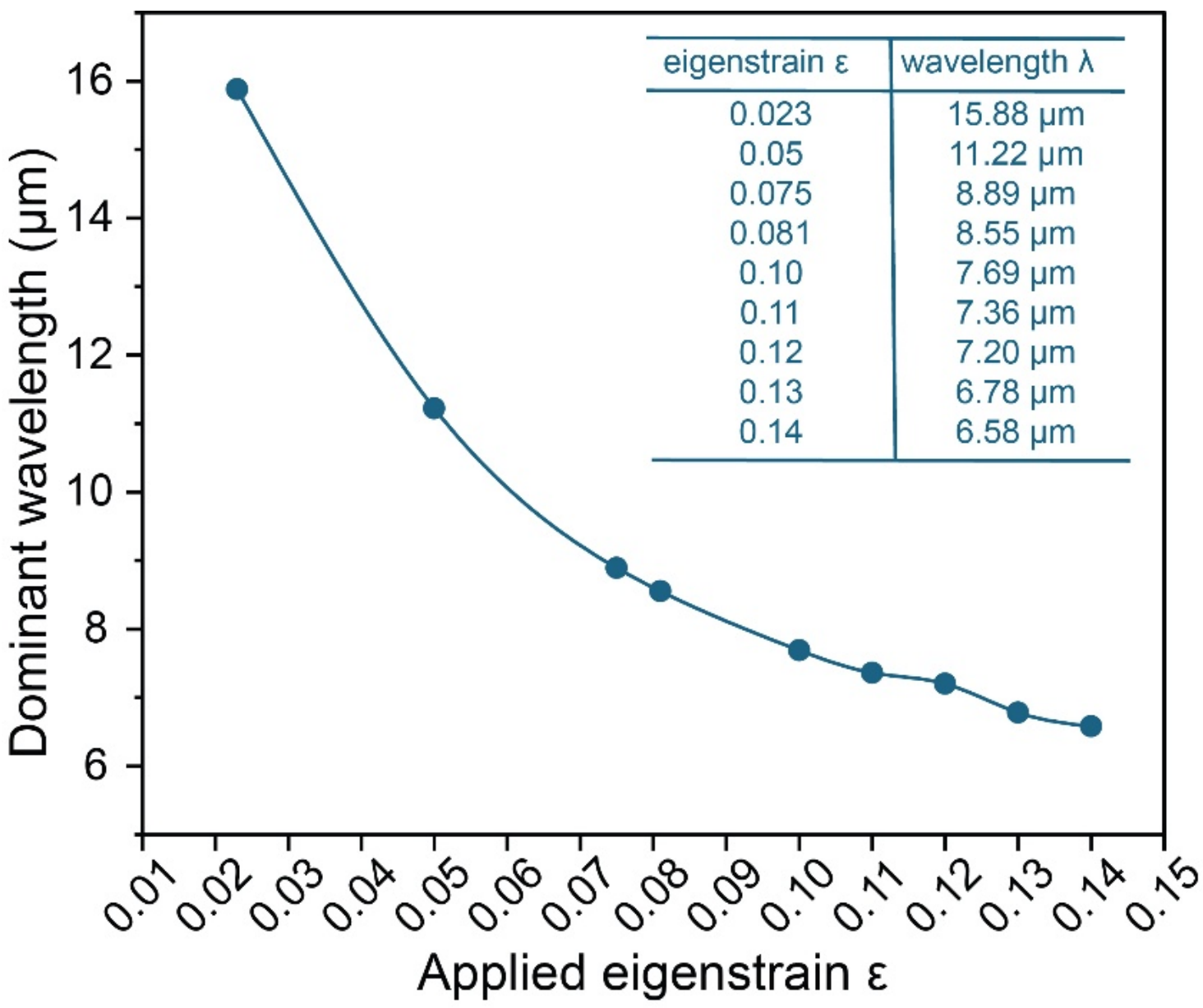

Figure. S4. Dominant wrinkling wavelength λ as a function of the eigenstrain ε. Similar to the experimental data, a larger eigenstrain indicates higher spore coverage, which leads to a smaller wrinkle wavelength.

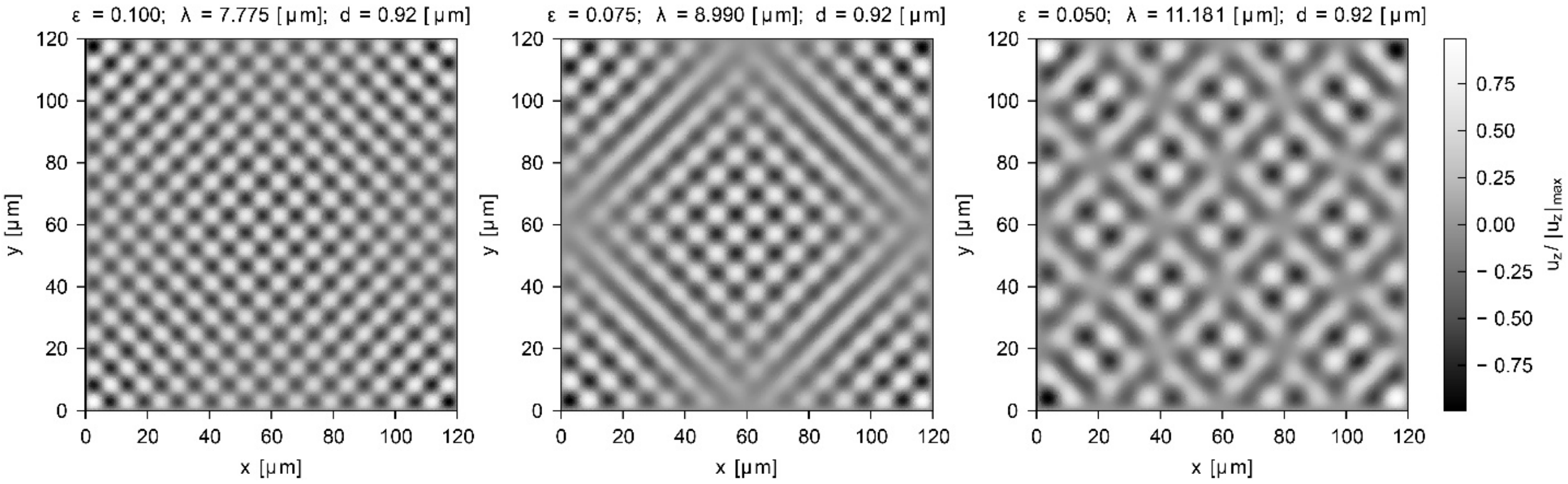

Figure. S5. Normalized out-of-plane displacement component of the critical wrinkling mode for increasing imposed eigenstrain ε.

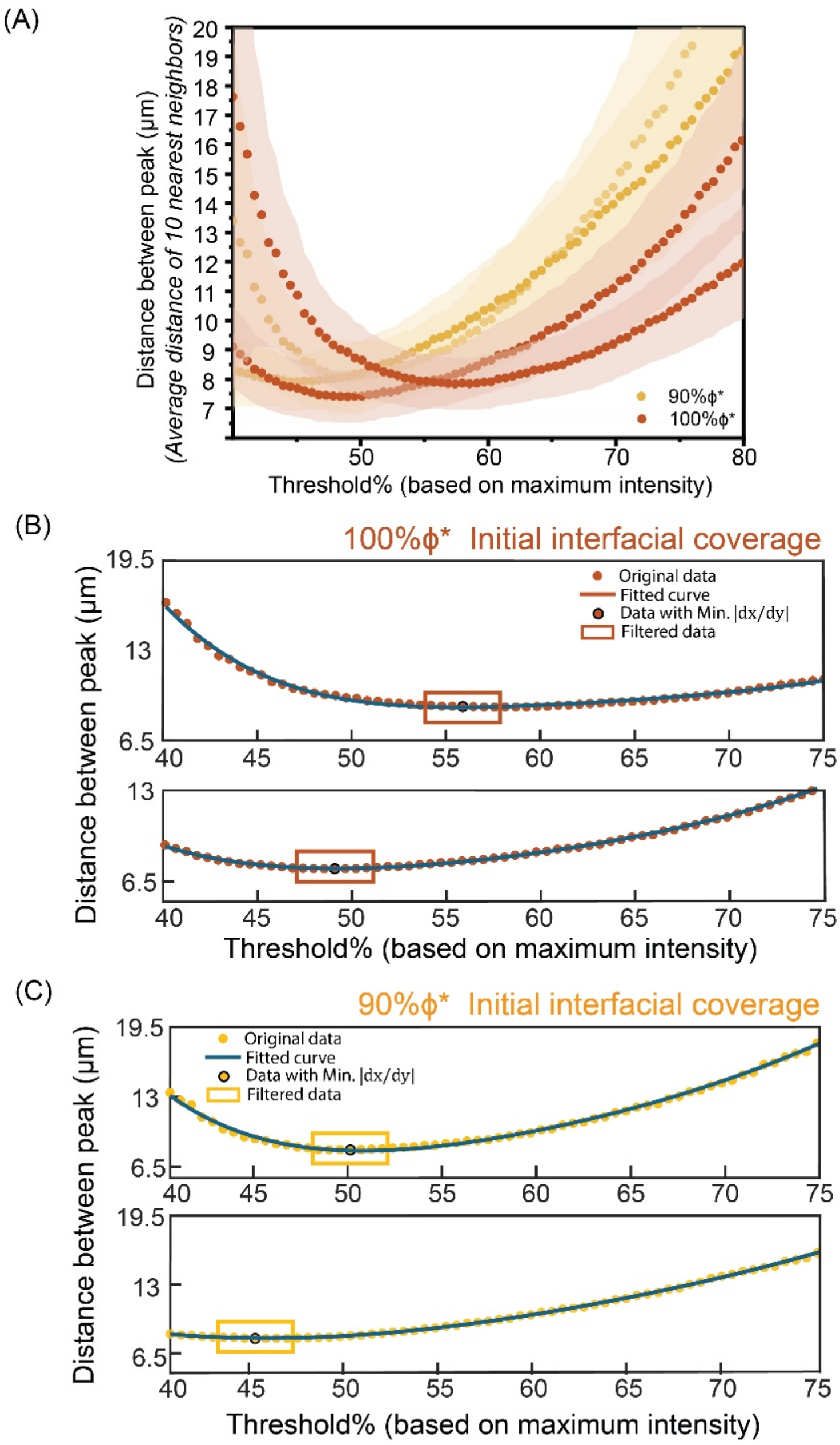

Figure. S6. Analysis of wrinkle peak distance. (A) Average distance of the 10 nearest peak neighbors in images applied with different threshold values. Threshold percentages are based on the maximum intensity of the normalized image, which is 255. As the threshold increased, the measured distance between peaks increased because wrinkles with smaller amplitudes, and thus lower intensities, were progressively filtered out. At very low threshold levels, noise significantly increased the measured distance. Two experimental replicates with 90%ϕ* and 100%ϕ* initial interfacial coverage were analyzed. **(B, C)** Zoomed-in curves for 100%ϕ* (B) and 90%ϕ* (C) initial interfacial coverage. Fits were based on fifth-order polynomial regression. 7 threshold value around the data with minimal absolute derivative was considered representative of the true wrinkle peaks, as the distance between peaks remained unaffected within this range.

Spores at TSB-Hexadecane interface at 4°C

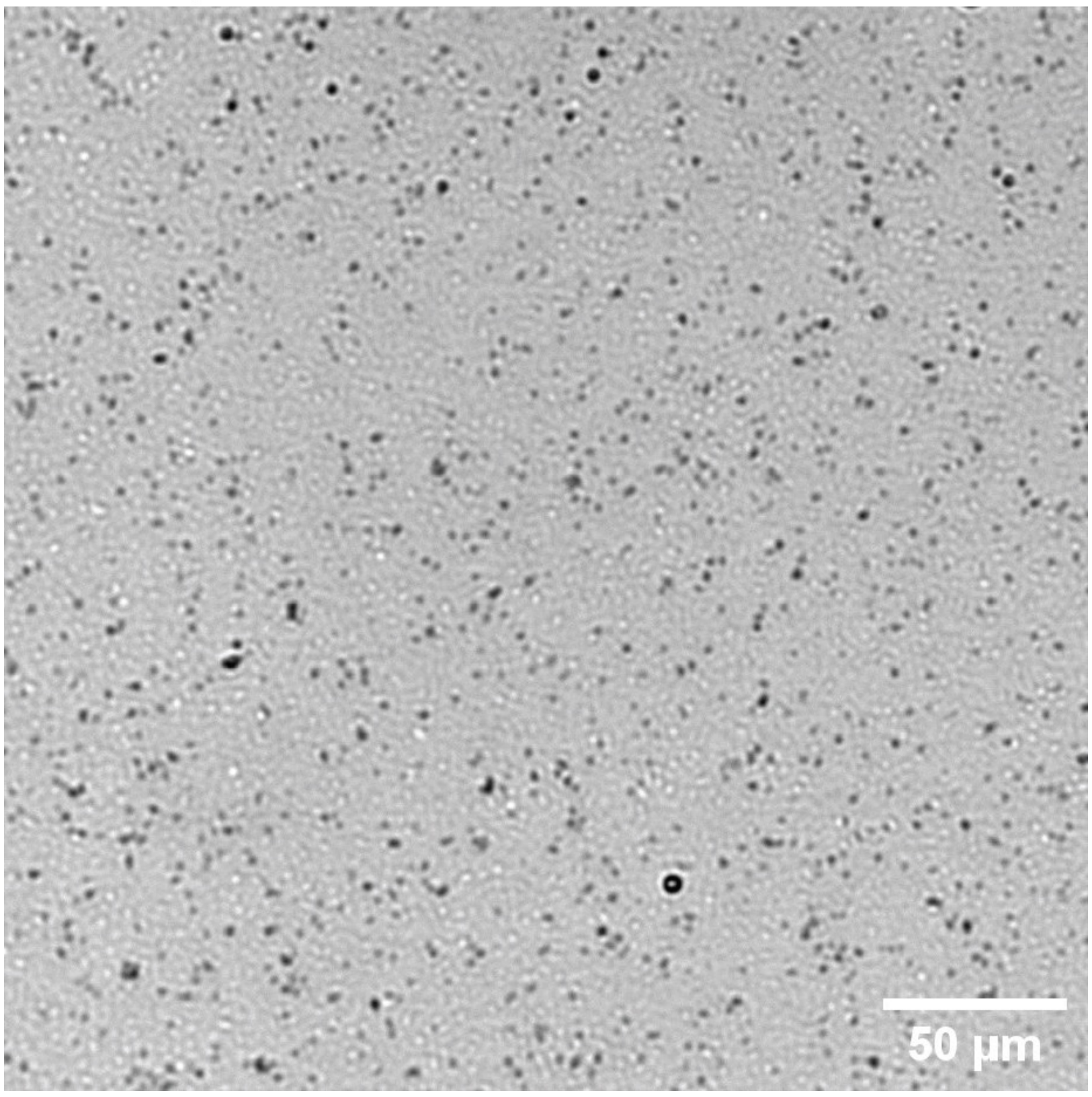

Figure. S7. Microscopy images of a spore monolayer at the hexadecane-TSB interface at 4 °C. No wrinkles were observed.

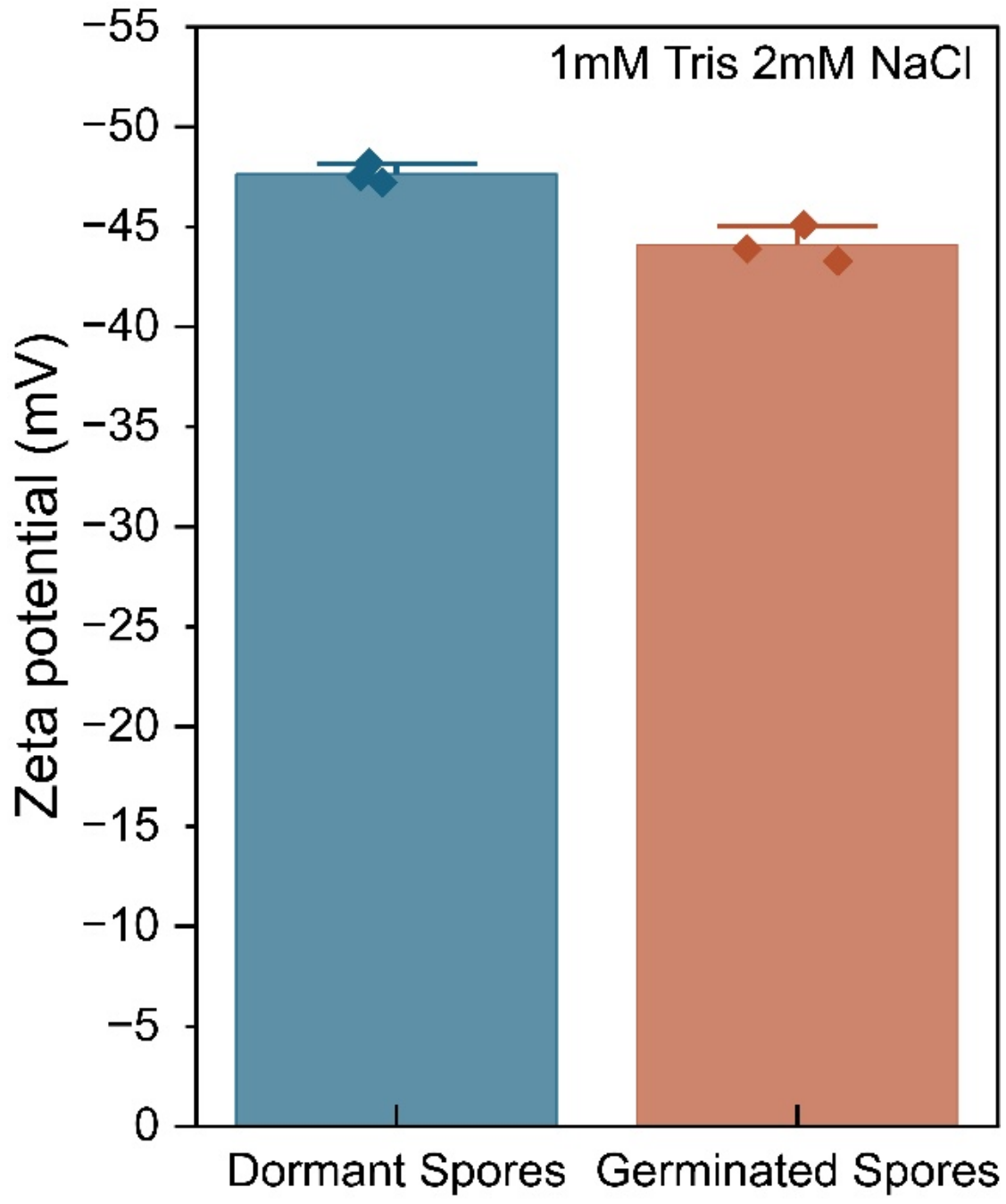

Figure. S8. Zeta potential of RFP168 dormant spores and the spores after germination in TSB for 40 mins. The measurements were performed in triplicate.

Movie S1.

Z-stack brightfield microscope images of a spore monolayer with 100%ϕ* initial interfacial coverage. The Z-stack was taken 108 mins after starting heating and adding the TSB. The images were taken every 2 µm.

Movie S2.

Timelapse of brightfield microscope images of a spore monolayer with 100%ϕ* initial interfacial coverage. The images were taken every 2 mins. Drift correction was applied to the timelapses.

Supplementary Text - *Wrinkling patterns due to Eigenstrain in elastic shells*

Flavio Lorez

To estimate the characteristic wrinkling wavelength, we model the system as a thin elastic shell of thickness d, and do a linear stability analysis on its tangent stiffness matrix. The effective Young's modulus of the shell is proportional to the interfacial surface tension γ and the monolayer thickness d via

$$E \approx 4.54\frac{\gamma}{d} \qquad (1)$$

where the numerical prefactor accounts for geometric Poisson effects and the solid fraction of circular particles arranged in hexagonal packing according to [1]. The surface tension γ of the water-oil interface is taken as $\gamma = 24.506$ mN/m; the thickness of the bacterial monolayer is estimated to be $d = 0.92\ \mu$m.

The Langmuir bath in the experiments is big enough such that boundary effects can be neglected when we look at a small central subdomain. Therefore, we model a periodic square RVE of side length $l = 120\ \mu$m. We apply periodic boundary conditions mapping the displacements and rotations on opposite edges of the domain ($\partial\Omega^-$ to $\partial\Omega^+$, see the schematic in **Figure. S8**). Bacterial growth is considered by an imposed isotropic compressive Eigenstrain ε. Furthermore, we consider the gravitational restoring force arising from the density difference between the water and oil phases, which is equivalent to a Winkler foundation.[2]

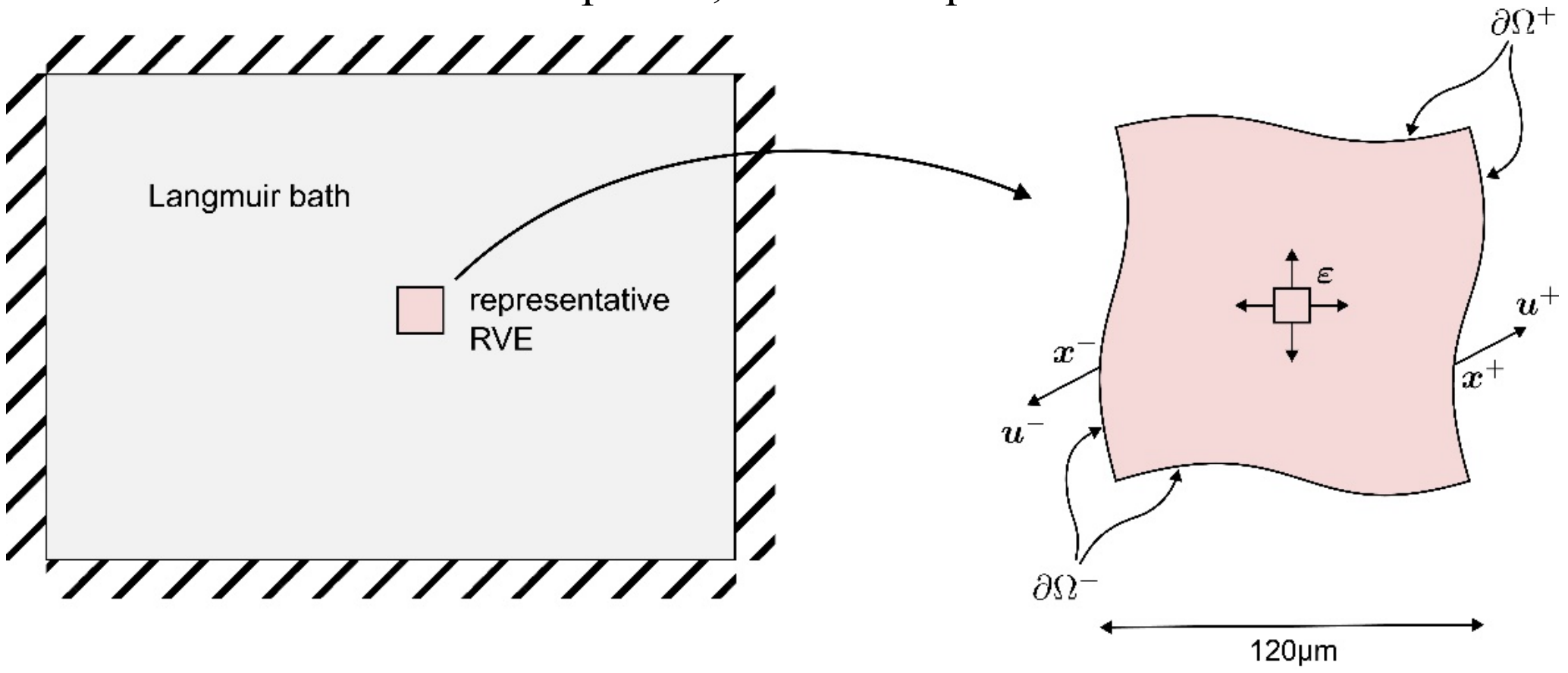

Figure. S8. Schematic of the modeled system. Periodic boundary conditions are applied on all sides of the square RVE of side length l = 120 μm, linking the bottom and top and left and right boundaries, respectively. u denotes the displacement and rotation degrees of freedom of the shell mid-surface. ε indicates the imposed isotropic compressive Eigenstrain due to spore germination.

The system is discretized using the finite element method (FEM). The shell kinematics are approximated using four-node quadrilateral MITC4 elements with five degrees of freedom per node. The degrees of freedom are denoted as u. A detailed discussion of the element formulation is omitted here; the reader is referred to standard references for theoretical and numerical details.[3–5] All simulations are performed using our in-house FEM framework.[6]

We formulate the problem in terms of a total potential energy functional as a function of the nodal degrees of freedom u and the imposed Eigenstrain ε,

$$\Pi(\mathrm{u}, \varepsilon) = \Pi_{mem}(\mathrm{u}, \varepsilon) + \Pi_{bend}(\mathrm{u}) + \Pi_{shear}(\mathrm{u}) + \Pi_{grav}(\mathrm{u}) \qquad (2)$$

where Π_{mem}, Π_{bend}, and Π_{shear} denote the membrane, bending, and transverse shear contributions of the shell, respectively, and Π_{grav} accounts for gravitational loading due to the

density mismatch across the interface. The Eigenstrain ε enters the formulation as a prestrain in the membrane energy term.

From the total energy functional, we compute the residual vector **r** and the consistent tangent stiffness matrix **K** via sparse automatic differentiation,

$$\mathrm{r}(\mathrm{u},\varepsilon) = \frac{\partial \Pi}{\partial \mathrm{u}}, \mathbf{K}(\mathrm{u},\varepsilon) = \frac{\partial^2 \Pi}{\partial u^2} \quad (3)$$

where u denotes the vector of nodal degrees of freedom, and assess linear stability by solving the generalized eigenvalue problem of the tangent stiffness matrix where u=0 and for a given Eigenstrain ε,

$$\mathbf{K}(u = 0, \varepsilon)\Phi = \Lambda\varphi \quad (4)$$

λ_{min} and its associated eigenmode Φ_{min} are considered to define the energy-minimizing wrinkling pattern of the spore monolayer, from which the characteristic wavelength is extracted by considering the FFT power spectrum of the out-of-plane displacement component of Φ_{min}.

REFERENCES

[1] D. Vella, P. Aussillous, L. Mahadevan, *EPL* **2004**, *68*, 212.
[2] D. A. Dillard, B. Mukherjee, P. Karnal, R. C. Batra, J. Frechette, *Soft Matter* **2018**, *14*, 3669.
[3] K.-J. Bathe, F. Brezzi, in *Numerical Techniques for Engineering Analysis and Design* (Eds.: G. N. Pande, J. Middleton), Springer Netherlands, Dordrecht, **1987**, pp. 407–417.
[4] Y. Ko, P.-S. Lee, K.-J. Bathe, *Computers & Structures* **2016**, *169*, 57.
[5] Y. Ko, Y. Lee, P.-S. Lee, K.-J. Bathe, *Computers & Structures* **2017**, *193*, 187.
[6] M. Pundir, F. Lorez, D. S. Kammer, **2026**, DOI 10.48550/arXiv.2602.12365.
